# Calibration of several first excited state properties for organic molecules through systematic comparison of TDDFT with experimental spectra†

**DOI:** 10.1039/d4tc03511a

**Published:** 2024-10-14

**Authors:** Xia Wu, Xiaoyu Xie, Alessandro Troisi

**Affiliations:** a Department of Chemistry, University of Liverpool Liverpool L69 3BX UK xiawu@liverpool.ac.uk A.Troisi@liverpool.ac.uk; b Qingdao Institute for Theoretical and Computational Sciences, School of Chemistry and Chemical Engineering, Shandong University Qingdao Shandong 266237 China xiaoyuxie@sdu.edu.cn

## Abstract

Time-dependent density functional theory (TDDFT) is a powerful computational tool for investigating excitation properties in organic electronics, and it holds significant potential for high-throughput virtual screening (HTVS) in this field. While most benchmarks focus on excitation energies, less attention has been paid to evaluating the accuracy of computed oscillator strengths and exciton reorganization energies against experimental data. In this work, we provide a systematic approach to evaluate in parallel the accuracy of these three quantities on the basis of a suitable fitting of the experimental absorption spectra of 71 molecules in solution. After considering 18 computational methodologies, the results from the M06-2X/def2-TZVP/PCM method demonstrate the strongest correlation with experimental data across the desired properties. For HTVS, the M06-2X/6-31G(d)/PCM method appears to be a particularly convenient choice among all methodologies due to its balance of computational efficiency and accuracy. Our results provide an additional benchmark needed before employing TDDFT methods for the discovery and design of organic electronic molecules.

## Abstract 2

We systematically evaluate computed excitation energies, oscillator strengths, and exciton reorganization energies by fitting experimental absorption spectra of 71 molecules.

## Graphical abstract

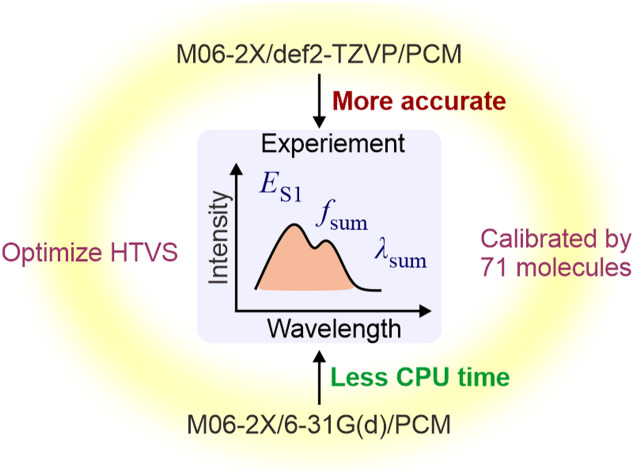

## Introduction

Time-dependent density functional theory (TDDFT) has gained significant traction in high-throughput virtual screening, enabling researchers to evaluate and predict the properties of molecules efficiently.^1–7^ Numerous studies have delved into the performance of TDDFT in calculating excitation energies, which is a crucial aspect of understanding the optical and electronic characteristics of materials.^8–19^ Naturally, the accuracy of these calculations needs to be thoroughly evaluated by comparing the computed excitation energies against experimental data.^20–23^ For example, Jacquemin and coworkers investigated the performance of various density functional theory methods in calculating the excitation energies of over 100 organic dyes from major chromophore classes.^24^ With an integrated design process involving theoretical calculations, machine learning, and experimental validation, highly efficient organic light-emitting diode molecules can be discovered from 1.6 million molecules with external quantum efficiencies up to 22%.^2^ In another recent example, a dataset of 48 182 organic semiconductors containing benchmarks of organic molecules was presented, providing relevant electronic properties and demonstrating its potential for repurposing known molecules.^25^ Thus, TDDFT provides a computationally efficient approach to modelling excited-state energies, facilitating the exploration of potential candidates for applications such as organic light-emitting diodes,^26,27^ photovoltaics,^28,29^ and photocatalysis.^30,31^

Nevertheless, for organic electronics, achieving optimal performance goes beyond merely aligning energy levels. In the absence of competing photophysical processes, the oscillator strength (*f*) determines the rate of light absorption and is strongly correlated with the efficiency of light emission, both of which are critical aspects in the performance of optoelectronic devices.^32^ Additionally, the exciton reorganization energy (*λ*), a measure of the structural changes accompanying the formation or dissociation of excitons, significantly influences phenomena such as exciton transport, emission efficiency and narrowness of absorption/emission peaks.^33–35^ Tailoring these properties through molecular design or external manipulation becomes imperative for applications such as organic light-emitting diodes, organic photovoltaics, and organic field-effect transistors, where efficient light-matter interactions and charge carrier mobilities are paramount.

Over the decade, numerous studies have benchmarked oscillator strengths using various functionals and basis sets.^36–39^ Recently, a few studies appeared that explore the accuracy of the computed oscillator strength of organic molecules in comparison to experimental data. In Cole's work, the oscillator strength is discussed as a computational property analogous to the experimental extinction coefficient, and they also analyzed the agreement between sTDA/TDDFT calculated *f* values and experimental intensity values.^40^ Gozem's team fitted and integrated 100 experimental UV-visible spectra of solvated organic molecules to obtain experimental oscillator strengths (*f*_exp_.), compared them to computed oscillator strengths (*f*_comp_.) from TDDFT, finding that while *f*_comp_. overestimates *f*_exp_., there is good overall correlation between the experimental and computed values.^41^ Alipour and coworkers explored the use of the optimally tuned range-separated hybrid density functionals combined with a polarizable continuum model (OT-RSHs-PCM) and their screened versions (OT-SRSHs-PCM) to reliably predict oscillator strengths for organic compounds.^42^ To the best of our knowledge, no prior studies have investigated the impact of *λ* with experimental data in the context of high-throughput virtual screening (HTVS), despite its crucial importance.

It is important to note that highly accurate methodologies have been developed over the years to reproduce the finest details of the vibronically-resolved spectra, including the effect of solvent,^43–45^ Duschinsky effect,^46,47^ and some of these results in publicly available software like FCClasses and ezFCF.^48,49^ Santoro and coworkers presented a mixed quantum-classical approach for computing vibronic absorption spectra of molecular aggregates, reproducing changes in absorption spectrum upon aggregation.^50^ Thiel's team analysed vertical and adiabatic Franck–Condon approaches using time-independent or time-dependent methods to simulate absorption spectra of three flavin compounds, finding that VFC with IMDHO-FA provides superior accuracy in spectral predictions.^51^ These studies, while suitable for a few molecules at a time, often require selecting the most appropriate quantum chemistry methodology. Consequently, they are not particularly suitable for comprehensive comparisons across a large set of molecules. Also, it is common for the work focusing on vibronic coupling to ignore energy level accuracy and oscillator strength.^52–54^ (there are also a large number of works comparing a range of quantum chemical methods among themselves and with experimental vertical excitation energy).^20–25^ These can be considered complementary to the work presented here, where we instead focus on a restricted number of methods popular in HTVS.

For TDDFT to be an effective screening protocol, it is crucial to evaluate the accuracy of all three components (excitation energy, *f*, and *λ*) *simultaneously*. This comprehensive assessment becomes particularly important when dealing with a large number of compounds, as one typically aims to employ a single efficient method for the screening process. HTVS involves in principle millions of molecules, making it impractical to select the optimal method for each dye individually. Moreover, when the exciton–phonon coupling is very strong (large *λ*), it becomes challenging to accurately extract the oscillator strength *f* without fitting the vibronic side peaks, which are affected by *λ*. In other words, it is not only useful but also more accurate to assess *f* and *λ* at the same time. In this work, we analysed 71 experimental absorption spectra and developed a fitting procedure to extract the excitation energy of the lowest excited state S_1_ (*E*_S1_), the corresponding oscillator strength (*f*_S1_) and *λ*. Subsequently, we compared the parameters derived experimentally and those computed with 18 different calculation levels to determine the most convenient approach and its expected accuracy.

## Methods

Usually, there are vibronic peaks for the S_1_ absorption spectrum. The intensity of these peaks can be evaluated as1
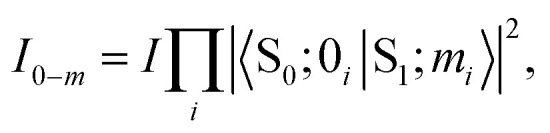
here, *m* = (*m*_1_, *m*_2_,…, *m*_*N*_) contains occupation numbers of *N* modes, |S_*k*_; *m*_*i*_〉 is a vibronic state for mode *i* at S_*k*_ potential energy surface state with *m*_*i*_ being the occupation number and *I* is the total intensity of S_1_ states, which is determined by its oscillator strength.

Under the harmonic approximation and Condon approximation, overlap in eqn (1) can be expressed as:2
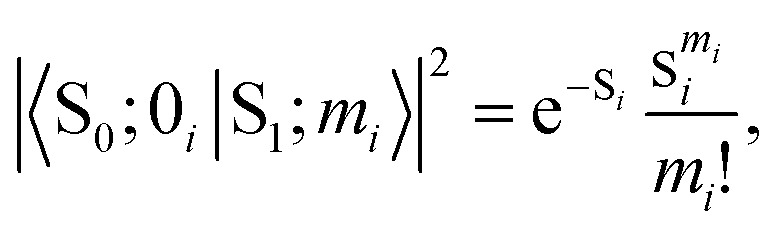
here, *s*_*i*_ is the Huang–Rhys (HR) factor of normal mode *i*, measuring the displacement of the excited state along that particular mode.^55^ Moreover, the product in eqn (1) can be further approximated as one overlap using one effective mode with HR factor *s* and vibrational frequency *ω*.

**Fig. 1:**
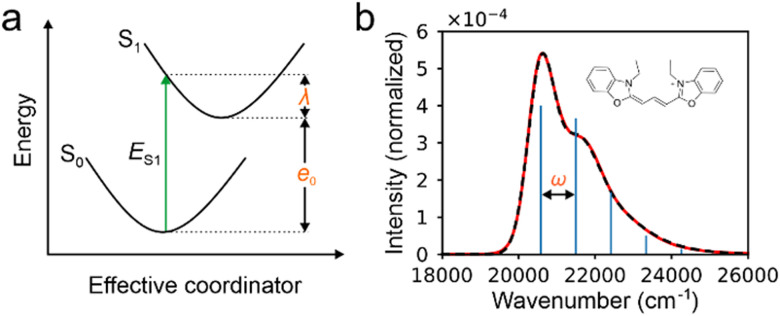
(a) Illustration of the relationship among the energy of the first excited state (*E*_S_1__), the reorganization energy (*λ*) and adiabatic excitation energy (*e*_0_ in the (one mode) model used to fit the experimental spectra of absorption only. (b) The experiment (the red line) and fitted absorption spectra (the dashed black line) for the molecule in the inset as example, resulting *e*_0_ = 20573.63 cm^−1^, *ω* = 920.38, *s* = 0.91, *σ*_0_ = 345.574 cm^−1^, Δ*σ* = 207.24 cm^−1^. The blue bars represent positions and intensities of vibrational peaks before broadening.

Then, the total spectrum can be decomposed as a set of normal distributions with centres *e*_0−*m*_ being,

3 *e*_0−*m*_ = *e*_0_ + *m*_*ω*_,

and intensities *I*_0−*m*_ being,4
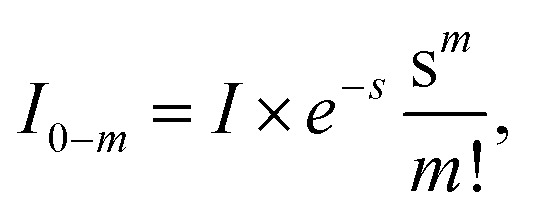
where *e*_0_ is the 0–0 transition energy (or adiabatic excitation energy) between S_1_ and S_0_, and *m* is the vibrational quantum number of the effective mode. For the broadening *σ*_0−*m*_ of normal distribution, two additional parameters are included considering the broadening of the pure electronic part *σ*_0_ and vibrational part Δ*σ*,

5 *σ*_0−*m*_ = *σ*_0_ + *m*Δ*σ*,

Therefore, there are six parameters for the spectrum fitting: adiabatic excitation energy (*e*_0_), frequency of the effective mode (*ω*), HR factor of the effective mode (*s*), the total intensity of S_1_ absorption (*I*) and two broadening parameters *σ*_0_ and Δ*σ* (see Fig. 1 for the illustration of the fitting), and the total fitting absorption spectrum is given by,6
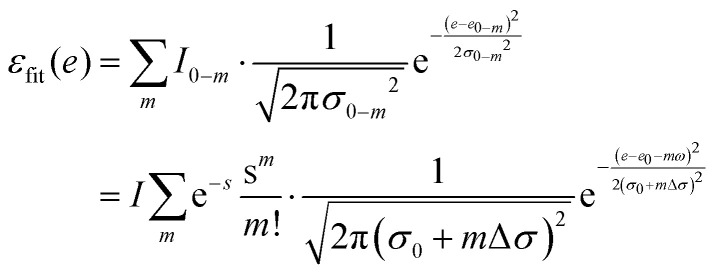


In practice, the sum over occupation number is truncated to 4 (*m* < 5), considering that HR factors of organic molecules are usually small.

After the fitting, oscillator strength can be calculated using the total intensity *I* obtained by fitting,^32^7
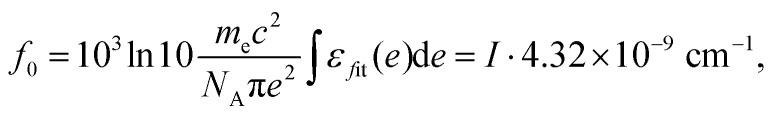
Here, *m*_*e*_ and *e* is the mass and charge of electron, respectively; *c* is speed of light in vacuum and *N*_A_ is the Avogadro's number.

However, since the experiments were conducted in different solvents, we incorporated the refractive index of the solvent (*n*) when fitting *f* by employing an alternative equation for more accurate calibration results, as suggested by the,ref. 41,42,56 and 57

8 *f*_S_1__ = *nf*_0_.

In general, the adiabatic energy differs from 0–0 energy depending on whether the zero-point energies (ZPE) in the S_0_ and S_1_ states are considered. However, our model (displaced harmonic oscillator) assumes the same curvature of the potential energy surfaces in the ground and excited states, resulting in identical ZPE. Consequently, within our model, the adiabatic energy and 0–0 energy are the same. The actual difference between ZPE was verified for few selected molecules reported in Table S4 of the ESI.† ^58^

In this work, the reorganization energy is defined as the difference between the energy of the vertical excitation from the equilibrium energy of S_0_ state and the lowest energy of the potential energy of S_1_ state. Thus, the exciton reorganization energy can be calculated by

9 *λ* = *sω*.

If we could fit a more complex vibronic model from the experiment, we could extract a more accurate experimental reorganization energy. An example of benchmark work considering of this type (not focused on organic electronics) was given in ref. 51. However, the low-resolution spectra we have prevent us from deriving such a model.

Then, the vertical excitation energy of S_1_ state can be evaluated as

10 *E*_S_1__ = *e*_0_ + *λ*.

The error of the fitting was computed as:11
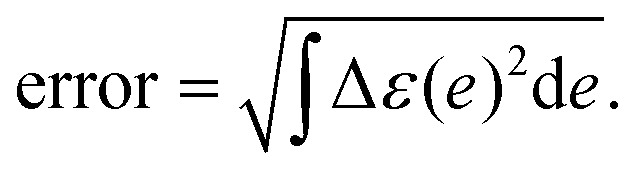


The Δ*ε*(*e*) represents the difference between experimental and fitting absorption spectrum (normalized).

The code for the fitting processes is now shared in GitHub by https://github.com/XiaoyuUoL/spectrum_fitting.

The UV/Vis^+^ photochemistry database of dyes is used in this work to investigate the performance of our fitting.^59^ All experimental data in the database are sourced from a single laboratory, with detailed experimental conditions to ensure consistency and avoid any potential instrumental errors. 103 dyes from the database with isolated S_1_ absorption peak or mirrored absorption/emission signal are selected for simultaneously evaluating of *e*_0_, *f*_S_1__ and *λ*. Besides, the spectra of the database were recorded in different solvents including ethylene glycol (*n* = 1.43), ethanol (*n* = 1.36), cyclohexane (*n* = 1.43), methanol (*n* = 1.33) and dimethyl sulfoxide (*n* = 1.48). The corresponding *n* was used for the fitting of *f*_S_1__, and the specific solvent was employed in the calculations including solvent effects. Since the original dataset focuses on dyes with relatively high oscillator strength, to improve its representativeness, several organic systems (named 061, 068, 077, 085, and 099) with low UV/Vis intensity are also collected from ref. 41 We use the names as given in the original database with full chemical detail provided in the ESI.†

To achieve more accurate fitting results, we select 71 molecules for the final dataset, each with an error below 0.03 cm^0.5^. This selection includes 66 molecules from the original 103 dyes and 5 additional molecules with low UV/Vis intensity (the molecular structures are shown in Fig. S1, ESI†). All results of fitting are presented in Table S1 (ESI†), Fig. 1b and Fig. S13–S82 (ESI†). The molecules with poor fitted results are almost invariably affected by the presence of a nearby excited state. Therefore, we believe there is no significant bias in excluding these molecules. Moreover, we verified that the S_2_ state in 71 molecules is mostly “dark” with much lower *f* values than the S_1_ state, and the energy gap between the S_1_ state and the next “bright” state exceeds 1 eV for most dyes, resulting no overlap between states for the dyes in our study.

All DFT and TDDFT calculations were carried out using the Gaussian 16A suite of programs.^60^ Geometry optimises 10 conformers using PM7 semi-empirical method. For the lowest energy PM7 conformer, geometry optimizations were carried out at the BLYP35/3-21G(d) level.^4^ Corresponding TDDFT calculations were utilizing the B3LYP, M06-2X, and ωB97XD functionals in conjunction with the 6-31G(d), def2-SVP, and def2-TZVP basis sets, total 18 methods. Solvation effects were all taken into account based on the polarizable continuum model (PCM), using the solvent as the same as that employed in corresponding experiments (see Table S1, ESI†).^61^ Gauge-invariance corrections are not included in our calculations. Due to stability issues, we didn’t consider diffuse functions which are not suitable for HTVS, where the goal is to achieve a high yield of converged calculations without human intervention. The molecular excitation properties were also investigated by the hole–electron analysis using Multiwfn 3.6.^62^ For the calculation of *λ*, we obtained results for 70 dyes, excluding the dye named ‘bfl’ (details in Fig. S1, ESI†) due to unsuccessful convergence in the S_1_ excited state.

## Results and discussion

**Fig. 2:**
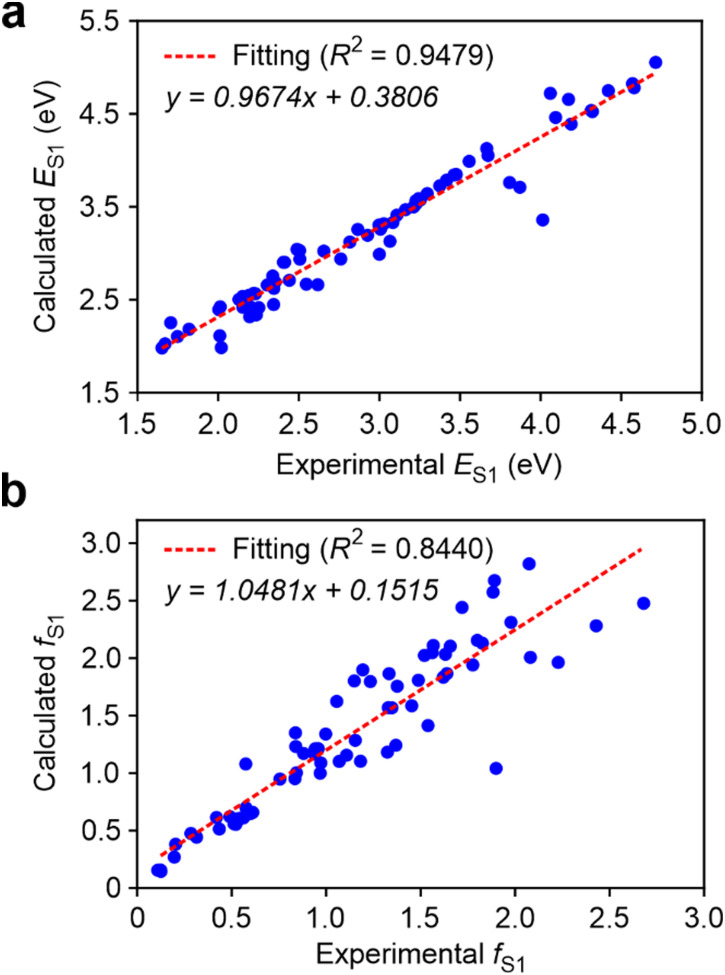
The correlation between the experimental fitted and calculated (a) the first vertical excited state energy *E*_S_1__ and (b) the corresponding oscillator strength *f*_S_1__ under M06-2X/def2-TZVP/PCM.

To begin, we computed *E*_S_1__ of 71 dyes in the dataset along with *f*_S_1__ using a total of 18 calculation levels (Table S2 and Fig. S2–S7, ESI†). The best results are obtained with the functional M06-2X in conjunction with the larger basis set (def2-TZVP), accounting for solvent effects. The calculated values for both *E*_S_1__ and *f*_S_1__ demonstrated a remarkable consistency with the experimental fitted data, yielding the coefficient of determination (*R*^2^) values of 0.948 and 0.844, respectively (Fig. 2a and b). Moreover, their slope of the linear fitted by M06-2X/def2-TZVP/PCM was both close to 1 (0.967 and 1.048, separately), indicating this method exhibit excellent accuracy in predicting *E*_S_1__ and *f*_S_1__. Computed *f*_S_1__ also exhibits a good correlation with experimental fitted *f*_0_ (*R*^2^ = 0.86), but the slope of the correlation is approximate 1.5 (Fig. S8, ESI†). This discrepancy prompted us to use *nf*_exp_. instead of *f*_exp_. to mitigate the inaccuracy as proposed in,ref. 33,34 and 36 where *n* is the refractive index of the solution.

**Fig. 3:**
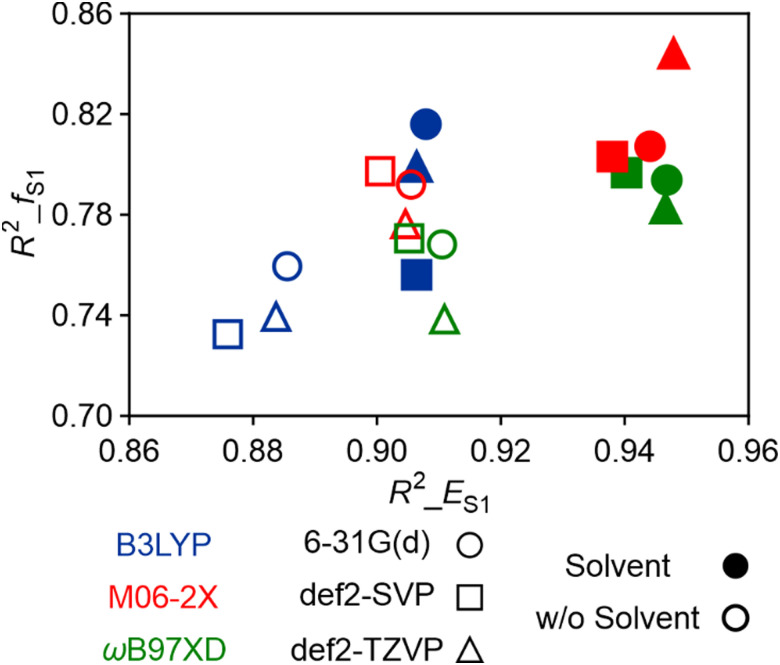
*R* ^2^ values of *E*_S_1__ and *f*_S_1__ between the experimental fitted and calculated data under 18 computational methods.

To illustrate the relative significance of functionals, basis sets, and solvent effects, we compared the results of 18 methods in Fig. 3. The choice of functional is crucial, as M06-2X and ωB97XD functionals yielded similar and superior performance compared to B3LYP, with the *R*^2^ exceeding 0.9 for both functionals. Basis set selection influences the results moderately, but its impact is less important. A more cost-effective calculation with the 6-31G(d) basis set can be recommended. Notably, it is noteworthy that the solvent effects are essential for accurately predicting both the *E*_S_1__ and the corresponding *f*_S_1__. The calculated values obtained using the PCM model exhibit noticeably better agreement with experimental data compared to gas-phase calculations employing the same functional. However, the impact of solvent effects is more pronounced on the calculations of *E*_S_1__ compared to *f*_S_1__.

Regarding *λ*, which involves the expensive geometry optimization of the S_1_ state, our initial approach utilized the M06-2X/6-31G(d) method to expedite the calculations, as its correlation for *E*_S_1__ and *f*_S_1__ was deemed acceptable. However, the obtained results for *λ* proved unsatisfactory, with an *R*^2^ of only 0.344 (Fig. S9a, ESI†). This poor performance can be primarily attributed to the inaccurate predictions for charged molecules, where the computed values deviated significantly from the experimental fitted data. Furthermore, even for few neutral molecules, the results exhibited large errors, further decreasing the *R*^2^ value for *λ*.

**Fig. 4:**
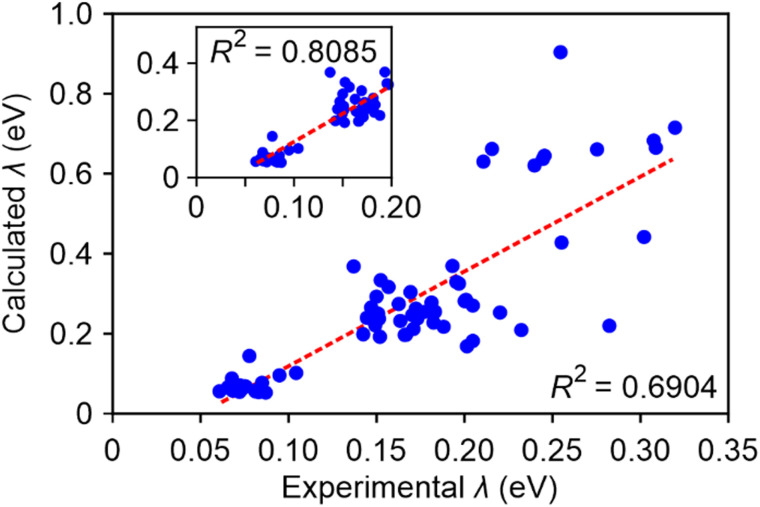
The correlation between the experimental fitted and calculated *λ* using M06-2X/def2-TZVP/PCM (equation: *y* = 2.3690*x* − 0.1181). The inset shows the results for small experimental fitted *λ* at 0–0.2 eV region (equation: *y* = 1.9650*x* − 0.0712).

Consequently, we employed our best-performing method, M06-2X/def2-TZVP/PCM, and observed a favourable correlation between the calculated and the experimental fitted *λ*, with an *R*^2^ value of approximately 0.7 (Fig. 4). For charged molecules, the computed values also aligned well with the fitted line. Within the experimental fitted *λ* range of 0 to 0.2 eV, the correlation strength is further improved, yielding an *R*^2^ value of 0.809. This underscores the substantial impact of incorporating solvent effects on the accuracy of reorganization energy calculations. Additionally, it is worth noting that the theoretical values tended to overestimate the experimental results by a factor of approximately 2.

The unsatisfactory outcomes associated with the M06-2X/6-31G(d) method prompted us to investigate the solvent effect on our systems more closely. Firstly, to avoid possible state crossing during the process of S_1_ state optimization, we checked all the molecules and ensure their S_1_ state has the same character between the Franck–Condon state and adiabatic excited state with and without PCM. Fig. S9a (ESI†) highlights an intriguing observation: dyes such as dasbt, pry1, pry2, and the sty series (molecular details provided in the ESI†) exhibit notably low calculated *λ* compared to experimental fitted data without solvent effects. Interestingly, these molecules share a common characteristic – they are all charged species. However, when solvent effects are introduced into the calculations (Fig. S9b, ESI†), the results align more closely with the experimental data, suggesting a significant influence of charges on the system behaviour, especially for *λ*. This is illustrated more in detail in Table 1, showing that both pyr1 and sty6 exhibit similar trends under solvated and gas-phase conditions, with relative errors becoming much smaller when the effect of the solvent is included.

**Table 1:** The experimental fitted and calculated *λ* using different methods and corresponding relative CPU time during the computation

Dyes	Experimental *λ* (eV)	Methods	Calculated *λ* (eV)	Relative CPU time
pyr1	0.195	M06-2X/6-31G(d)	0.046	1.000
		M06-2X/def2-TZVP	0.044	13.894
		M06-2X/6-31G(d)/PCM	0.318	1.048
		M06-2X/def2-TZVP/PCM	0.329	13.095
sty6	0.205	M06-2X/6-31G(d)	0.050	1.503
		M06-2X/def2-TZVP	0.053	11.873
		M06-2X/6-31G(d)/PCM	0.161	1.926
		M06-2X/def2-TZVP/PCM	0.182	17.894

Nevertheless, certain charged molecules remain unaffected by solvent effects, as evidenced by their consistent placement near the fitting curve, irrespective of the presence or absence of solvent environments. Examples include rh6g and hidci, where the calculated *λ* exhibit good correlations with the experimental fitted *λ* regardless of the inclusion of solvent effects (Table S2, ESI†). Therefore, it becomes evident that the presence of charges alone does not directly influence the calculated *λ* results. To gain deeper insights, we investigated the distance between the centroid of the electron and hole distributions, as illustrated in Fig. S11 and Table S2 (ESI†). Molecules like pyr1 and sty6 exhibit a larger separation between the electron and hole centroids (larger than 2 Å) compared to rh6g and hidci (less than 2 Å). This characteristic of spatially separated charge distributions makes them more susceptible to the stabilizing effects of the solvent environment.

Table 1 also reports the relative computational costs by using four cores for the different methods employed, which are representative for molecules of these sizes. These sizes, in turn, are typical for molecules considered in virtual screening protocols. Clearly, the significantly improved accuracy of calculations for *λ* including the implicit solvent effect carries a very small additional computational cost. However, the inclusion of a large basis set does not offer a major advantage in accuracy, despite being substantially more expensive computationally. When considered alongside the results for the excitation energy and oscillator strength it seems that M06-2X/6-31G(d)/PCM offers among the best compromises between accuracy and speed across different quantities to evaluate.

## Conclusions

In summary, we proposed a procedure to fit automatically experimental absorption spectra of medium-sized organic molecules in solution and obtain parameters that can be accessed experimentally: the excitation energy of the first excited state, corresponding oscillator strength, and exciton reorganization energy. By comparing computed and experimental parameters for a dataset of 71 molecules we can determine the accuracy expected from a range of TDDFT methods routinely used in the digital design of molecules for organic electronics. Our study demonstrates a robust correlation between calculated and experimental S_1_ energy, with most *R*^2^ values exceeding 0.9, particularly achieving *R*^2^ = 0.948 with the M06-2X/def2-TZVP/PCM method. The same method also produces very good quality predictions of oscillator strength (*R*^2^ = 0.844) and reasonable predictions (*R*^2^ = 0.690) of the excitation reorganization energy (the quality of the latter prediction improves for technologically relevant dyes with reorganization energy < 0.2 eV). This suggests that the M06-2X/def2-TZVP/PCM method exhibits the best overall performance among the 18 computational methods tested. Comparison of several methodologies highlights that the inclusion of implicit solvent effects is essential to achieve good predictivity while extended basis sets are not as critical: the results obtained with the smaller 6-31G(d) basis set seems to be sufficiently accurate for high-throughput screening applications. These benchmarks can be used to determine the expected accuracy of computational predictions and to introduce suitable corrections to the systematic errors inherent to the computational methods. Our procedure will yield increasingly more accurate and informative results as larger and homogenous experimental data sets become available in the future.

## Data availability

The code for the fitting processes is now shared in GitHub by https://github.com/XiaoyuUoL/spectrum_fitting.

## Conflicts of interest

There are no conflicts to declare.

## Supplementary Material

TC-012-D4TC03511A-s001
