# Supplementary material for: Calibration of several first excited state properties for organic molecules through systematic comparison of TDDFT with experimental spectra
Source: J Mater Chem C Mater. 2024 Oct 14;12(46):18886–92. doi: 10.1039/d4tc03511a (PMC11492815; doi:10.1039/d4tc03511a)
Supplement: TC-012-D4TC03511A-s001 [file TC-012-D4TC03511A-s001.pdf]

Supplementary Information (SI) for Journal of Materials Chemistry C.
This journal is © The Royal Society of Chemistry 2024

Electronic Supplementary Information (ESI)

Calibration of Several First Excited State Properties for Organic Molecules through Systematic Comparison of TDDFT with Experimental Spectra

Xia Wu,*[a] Xiaoyu Xie *[b] and Alessandro Troisi *[a]

a. Department of Chemistry, University of Liverpool, Liverpool, L69 3BX, United Kingdom.

b. Qingdao Institute for Theoretical and Computational Sciences, School of Chemistry and Chemical Engineering, Shandong University, Qingdao, Shandong 266237, China.

* Email: xiawu@liverpool.ac.uk, xiaoyuxie@sdu.edu.cn, A.Troisi@liverpool.ac.uk

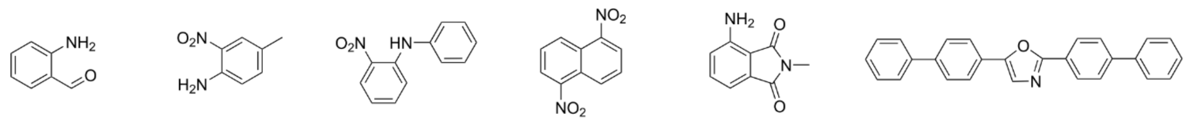

061 068 077 085 099 bbo

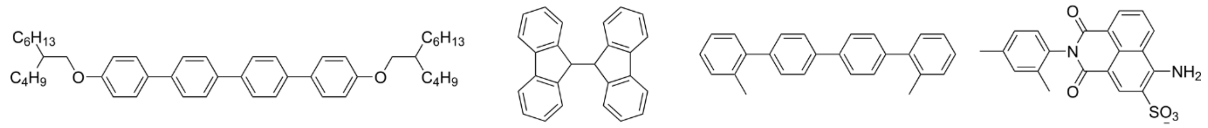

bbq bfl bmq bsf

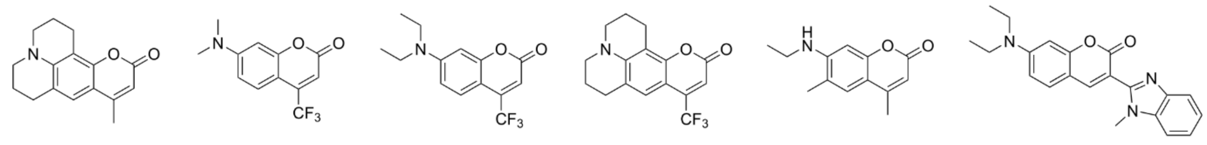

c102 c152 c152a c153 c2 c30

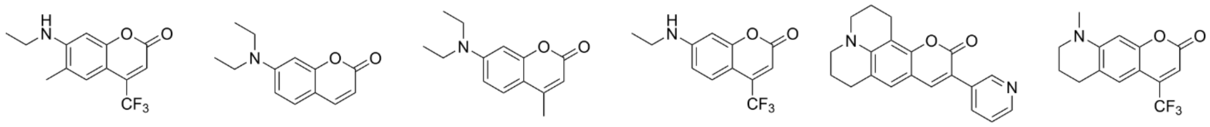

c307 c466 c47 c500 c510 c522

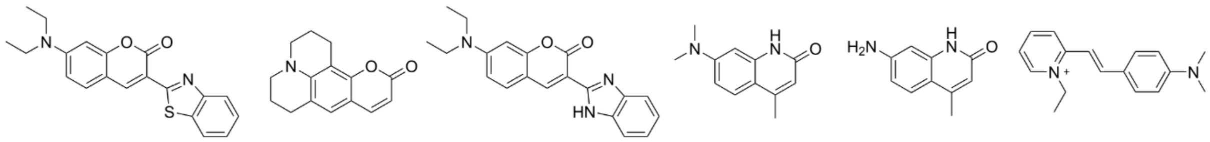

c6 c6h c7 cs3 cs7 daspi

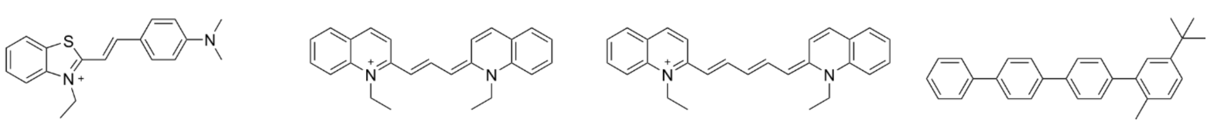

dasbt dci-2 ddi dmq

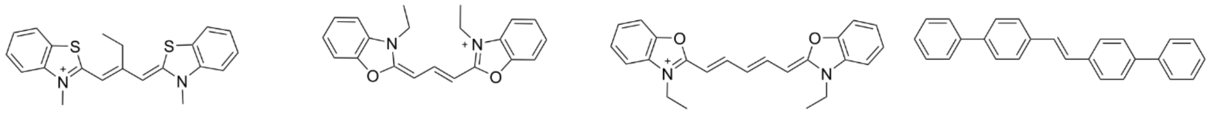

dmetc doci dodci dps

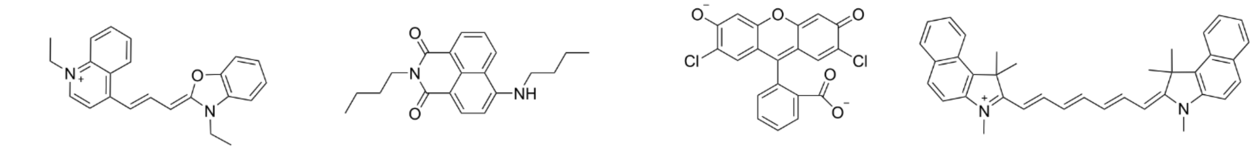

dqoci f7ga fl27 hditc

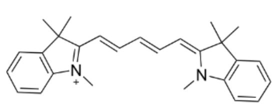

hidci

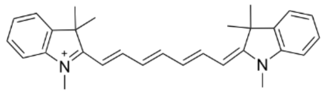

hitci

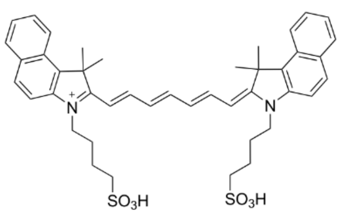

ir125

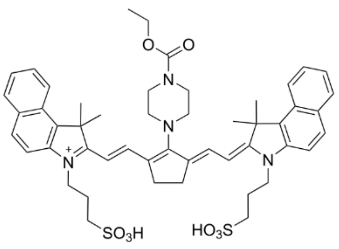

ir144

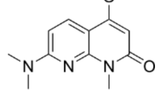

q390

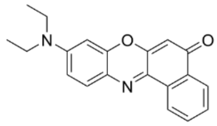

pheox

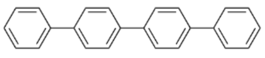

pqp

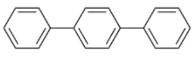

ptp

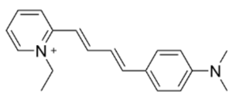

pyr1

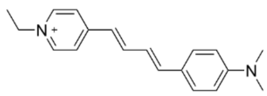

pyr2

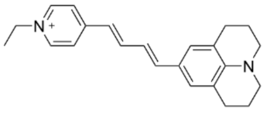

pyr4

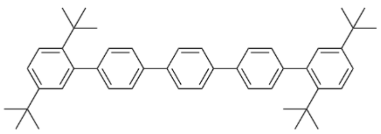

qui

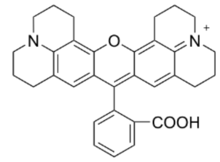

rh101

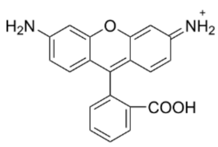

rh110

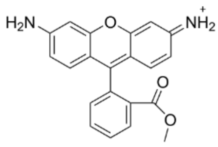

rh123

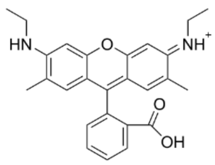

rh19a

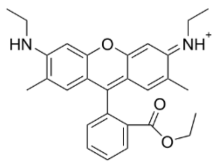

rh6g

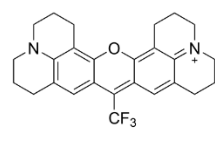

rh700

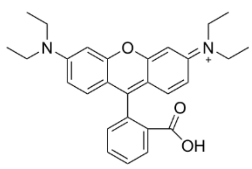

rhb

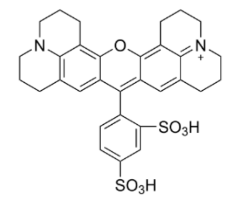

sr101

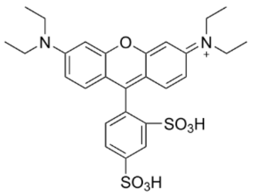

srhb

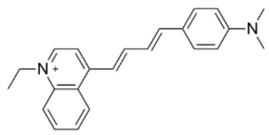

sty11

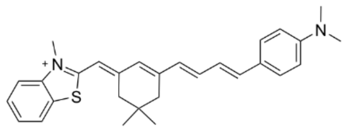

sty14

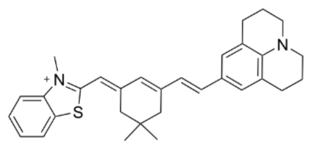

sty15

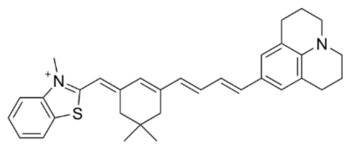

sty20

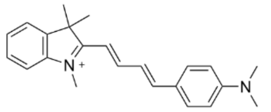

sty6

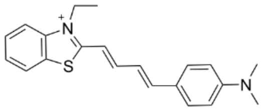

sty8

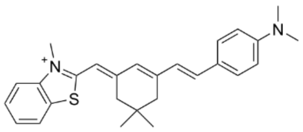

sty9

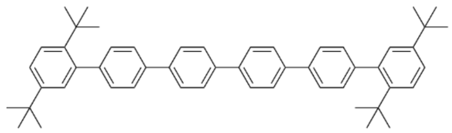

tbs

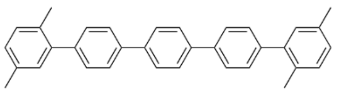

tmi

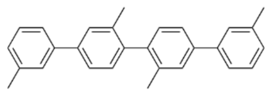

tmq

Figure S1. The molecule structures of 71 dyes in this work. The labels of the compounds are those used in the references given.

Table S1. The fitting details of all dyes in this work.

ID	Solvent	e_0 (cm^{-1})	ω (cm^{-1})	s	σ_0 (cm^{-1})	$\Delta\sigma$ (cm^{-1})	f_{exp}	$error$
061	Ethanol	26746.331	1913.952	0.609	1153.683	228.199	0.093	0.017
068	Methanol	23494.270	1890.654	0.650	1463.281	257.431	0.094	0.015
077	Methanol	23090.179	1994.296	0.554	1554.254	263.276	0.148	0.013
085	Methanol	29790.788	1968.035	1.310	1716.600	789.602	0.214	0.018
099	Ethanol	24979.799	1672.874	0.756	1098.153	138.596	0.080	0.022
bbo	Cyclohexane	28652.551	1765.056	1.166	1162.739	152.400	1.133	0.029
bbq	Cyclohexane	31854.710	1845.265	1.048	1443.540	309.570	1.383	0.018
bfl	Cyclohexane	30662.199	1936.038	1.076	1090.482	619.790	1.328	0.023
bmq	Cyclohexane	34380.467	1995.617	1.243	1354.415	3.850	0.930	0.012
bsf	Ethanol	22699.013	1424.953	1.037	888.987	31.420	0.150	0.009
c102	Ethanol	25212.296	1614.812	0.854	959.514	120.910	0.403	0.013
c152	Ethanol	24513.526	1703.809	0.820	1093.269	0.140	0.380	0.009
c152a	Ethanol	24281.173	1782.693	0.685	1124.459	19.920	0.414	0.013
c153	Ethanol	22900.659	1556.259	0.975	1022.427	79.120	0.388	0.020
c2	Ethanol	26579.770	1646.190	0.888	898.116	14.930	0.391	0.011
c30	Ethanol	23660.887	1431.507	0.845	867.288	80.830	0.705	0.015
c307	Ethanol	24376.170	1582.836	1.043	972.676	60.390	0.376	0.016
c466	Ethanol	25851.848	1529.049	0.893	905.606	50.630	0.423	0.007
c47	Ethanol	26127.239	1462.795	0.977	863.987	108.960	0.450	0.006
c500	Ethanol	24244.030	1501.247	1.183	919.847	50.170	0.384	0.007
c510	Ethanol	22783.887	1129.778	1.298	809.406	217.130	0.620	0.011
c522	Ethanol	23596.029	1616.518	0.910	975.210	0.800	0.390	0.010
c6	Ethanol	21379.352	1217.276	1.100	589.759	102.590	0.616	0.020
c6h	Ethanol	24814.115	1530.350	0.879	920.823	74.560	0.443	0.009
c7	Ethanol	21730.359	938.106	1.997	481.788	285.550	0.849	0.011
cs3	Ethanol	27538.454	1461.559	0.786	742.234	7.070	0.309	0.009
cs7	Ethanol	28378.867	1496.435	0.792	774.579	15.740	0.320	0.013
dasbt	Ethanol	18465.229	976.295	1.255	694.118	234.510	0.776	0.021
daspi	Ethanol	20873.819	1534.443	0.906	1081.111	242.820	0.734	0.019
dci-2	Ethanol	16484.222	1242.845	0.551	354.025	88.500	1.118	0.019
ddi	Ethanol	14082.220	1183.993	0.497	346.364	149.080	1.385	0.030
dmetc	Ethanol	18387.716	917.621	0.576	501.885	183.900	0.869	0.021
dmq	Cyclohexane	33425.552	1986.306	1.118	1344.982	55.560	1.016	0.010
doci	Ethanol	20573.635	920.379	0.914	345.547	207.240	1.012	0.013
dodci	Ethylene glycol	17050.104	745.956	0.813	348.457	381.410	1.699	0.020
dps	Cyclohexane	28787.023	1700.010	1.433	1125.132	358.260	1.455	0.026
dqoci	Ethanol	16690.441	573.715	1.167	327.236	223.660	0.991	0.023
f7ga	Ethanol	21910.547	1470.019	0.819	876.592	0.020	0.231	0.011
fl27	Methanol	19606.715	920.731	0.663	369.173	349.940	0.728	0.018
hditc	Ethanol	12771.064	832.393	0.843	325.135	378.890	1.525	0.019

hidci	Ethanol	15578.305	953.618	0.613	328.707	239.200	1.344	0.018
hitci	Ethanol	13440.777	841.202	0.777	337.023	433.010	1.390	0.026
ir125	Ethanol	12663.488	810.101	0.808	327.737	396.950	1.306	0.021
ir144	Ethanol	13130.198	839.164	0.747	595.261	232.390	1.199	0.015
pheox	Ethanol	17662.449	963.381	1.369	706.090	206.680	0.423	0.021
pqp	Cyclohexane	32868.423	1991.818	0.994	1406.662	101.840	1.040	0.012
ptp	Cyclohexane	34448.128	1846.001	1.349	1264.402	2.310	0.958	0.013
pyr1	Ethanol	19538.142	1866.698	0.844	1277.380	103.210	0.845	0.015
pyr2	Ethanol	19227.864	1984.359	0.661	1328.790	65.630	0.907	0.011
pyr4	Ethanol	17291.275	1550.152	1.041	1122.428	126.260	0.878	0.009
q390	Ethanol	28007.960	835.208	1.945	356.615	379.340	0.361	0.019
qui	Cyclohexane	31919.952	1725.159	1.008	1567.052	453.220	1.558	0.014
rh101	Ethanol	17411.416	990.776	0.587	374.126	303.880	0.691	0.019
rh110	Ethanol	19612.833	826.420	0.718	378.760	357.390	0.614	0.017
rh123	Ethanol	19527.726	872.175	0.624	381.142	340.870	0.556	0.018
rh19a	Methanol	18913.264	978.471	0.550	401.665	261.800	0.804	0.013
rh6g	Ethanol	18850.152	921.879	0.593	389.062	284.870	0.715	0.013
rh700	Ethanol	15467.184	1241.540	0.616	359.330	167.370	0.647	0.031
rhb	Ethanol	18369.244	1037.628	0.472	410.811	192.560	0.617	0.014
sr101	Ethanol	17360.413	1005.039	0.547	343.151	317.570	0.815	0.029
srhb	Ethanol	18035.036	998.930	0.552	372.471	260.310	0.693	0.024
sty11	Ethanol	16819.329	1957.404	0.622	1275.559	83.030	0.980	0.014
sty14	Ethanol	16146.458	1848.037	0.843	1543.531	256.080	1.264	0.016
sty15	Ethanol	13933.974	1484.501	1.534	879.402	134.780	0.974	0.010
sty20	Ethanol	14697.723	1926.908	0.824	1461.345	284.270	1.132	0.022
sty6	Ethanol	15707.763	996.461	1.657	600.898	317.520	1.218	0.012
sty8	Ethanol	16788.467	1760.925	0.780	953.988	15.660	1.152	0.023
sty9	Ethanol	16161.630	1997.636	0.811	1264.811	8.110	1.148	0.022
tbs	Cyclohexane	31313.246	1933.549	0.879	1490.848	421.250	1.874	0.019
tmi	Cyclohexane	32821.558	1851.815	1.065	1459.528	277.070	1.259	0.015
tmq	Cyclohexane	35965.135	1517.588	1.353	1692.059	118.360	1.146	0.023

Table S2. The correlation results between the experimental fitted and calculated E_{S1} and f_{S1} in different computational methods, respectively.

Computational methods			Exp. Vs. Cal.	
Functional	Basis Set	PCM (Y/N)	R^2 (E_{S1})	R^2 (f_{S1})
B3LYP	6-31G(d)	N	0.8855	0.7595
B3LYP	6-31G(d)	Y	0.9079	0.8160
B3LYP	def2-SVP	N	0.8759	0.7325
B3LYP	def2-SVP	Y	0.9064	0.7561
B3LYP	def2-TZVP	N	0.8837	0.7394
B3LYP	def2-TZVP	Y	0.9064	0.7990
M06-2X	6-31G(d)	N	0.9055	0.7920
M06-2X	6-31G(d)	Y	0.9441	0.8072
M06-2X	def2-SVP	N	0.9005	0.7973
M06-2X	def2-SVP	Y	0.9380	0.8031
M06-2X	def2-TZVP	N	0.9046	0.7763
M06-2X	def2-TZVP	Y	0.9479	0.8440
ωB97XD	6-31G(d)	N	0.9105	0.7682
ωB97XD	6-31G(d)	Y	0.9468	0.7938
ωB97XD	def2-SVP	N	0.9052	0.7712
ωB97XD	def2-SVP	Y	0.9403	0.7973
ωB97XD	def2-TZVP	N	0.9109	0.7390
ωB97XD	def2-TZVP	Y	0.9466	0.7830

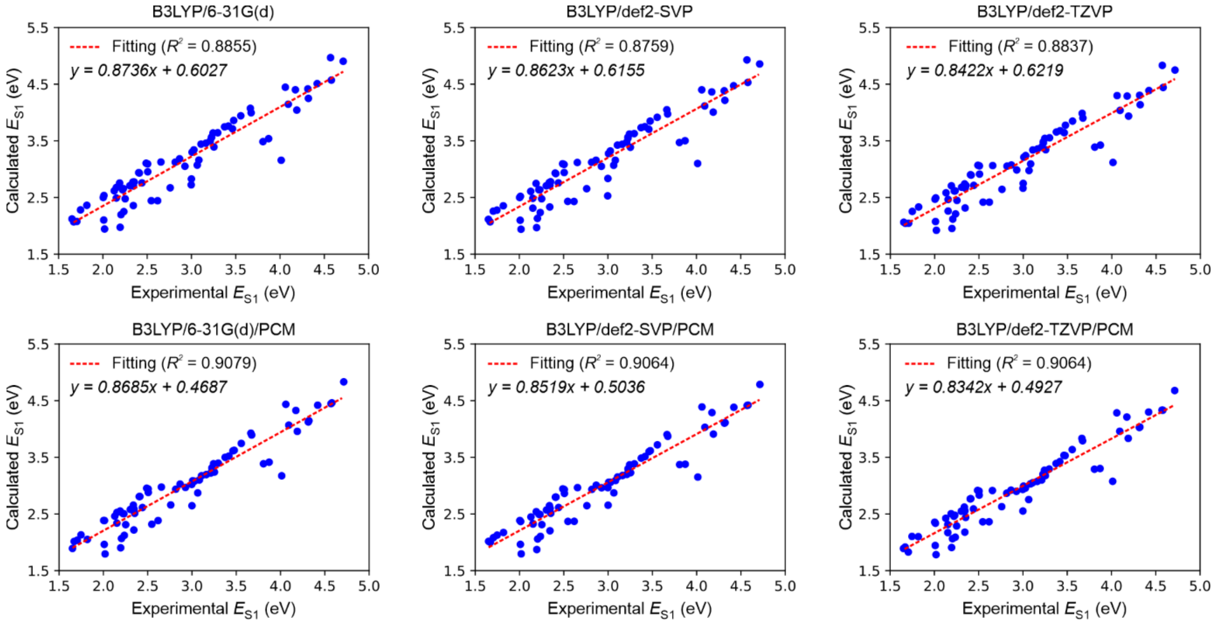

Figure S2. The correlation between the experimental fitted and calculated E_{S1} using the functional of B3LYP with different basic sets in gas phase or in solvents.

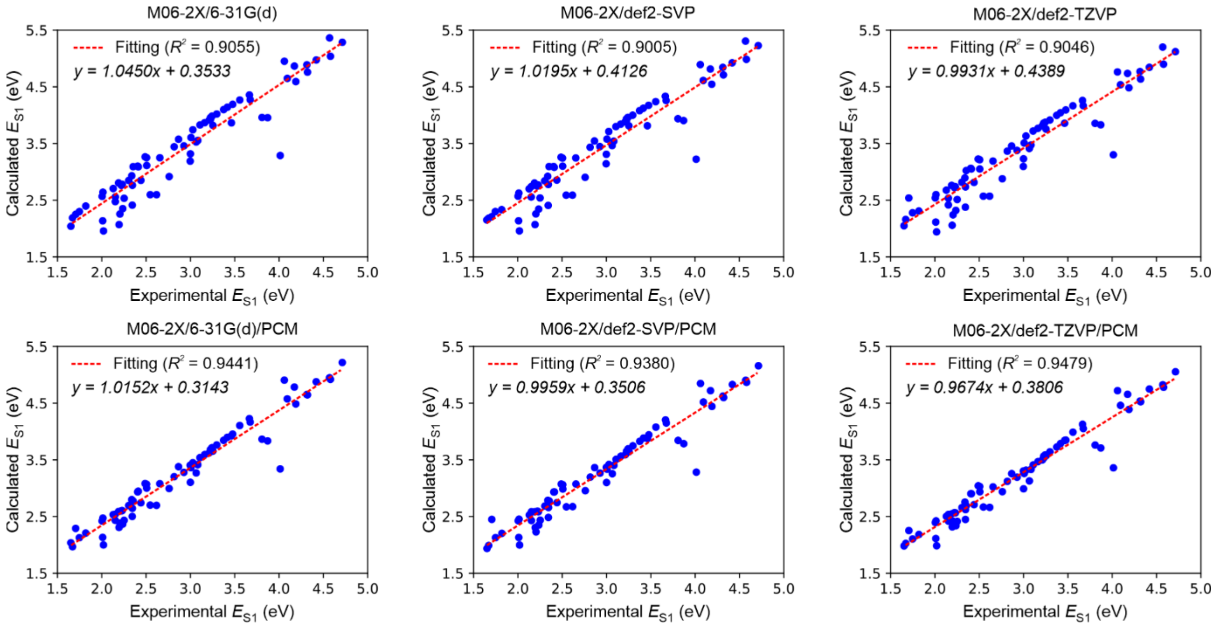

Figure S3. The correlation between the experimental fitted and calculated E_{S1} using the functional of M06-2X with different basic sets in gas phase or in solvents.

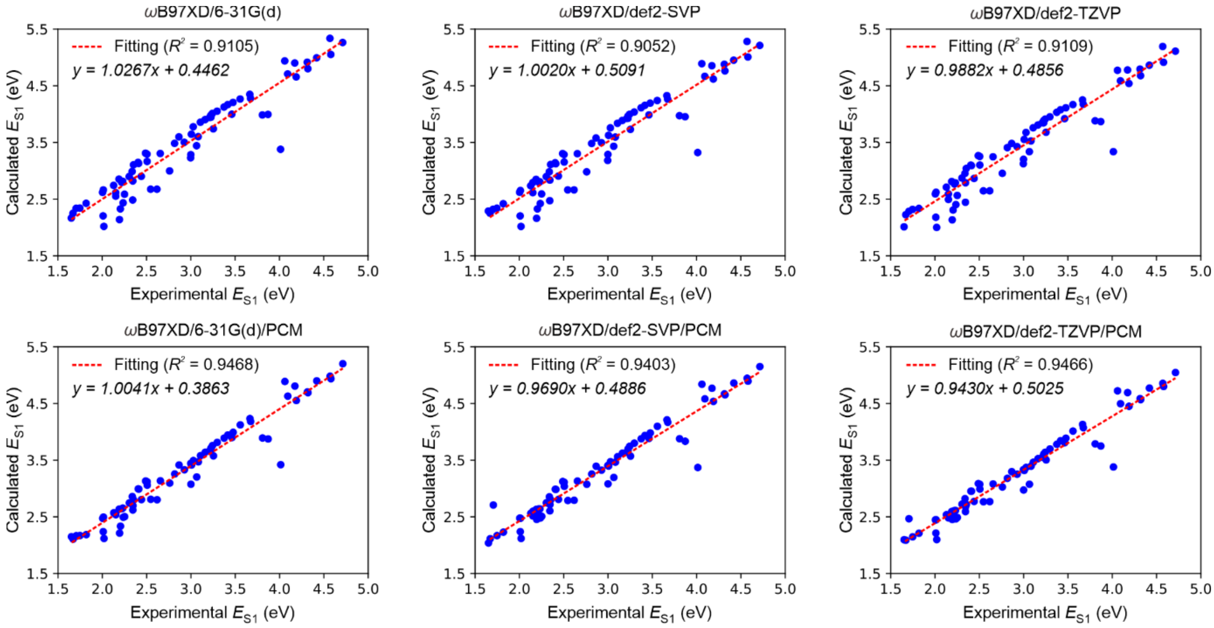

Figure S4. The correlation between the experimental fitted and calculated E_{S1} using the functional of ωB97XD with different basic sets in gas phase or in solvents.

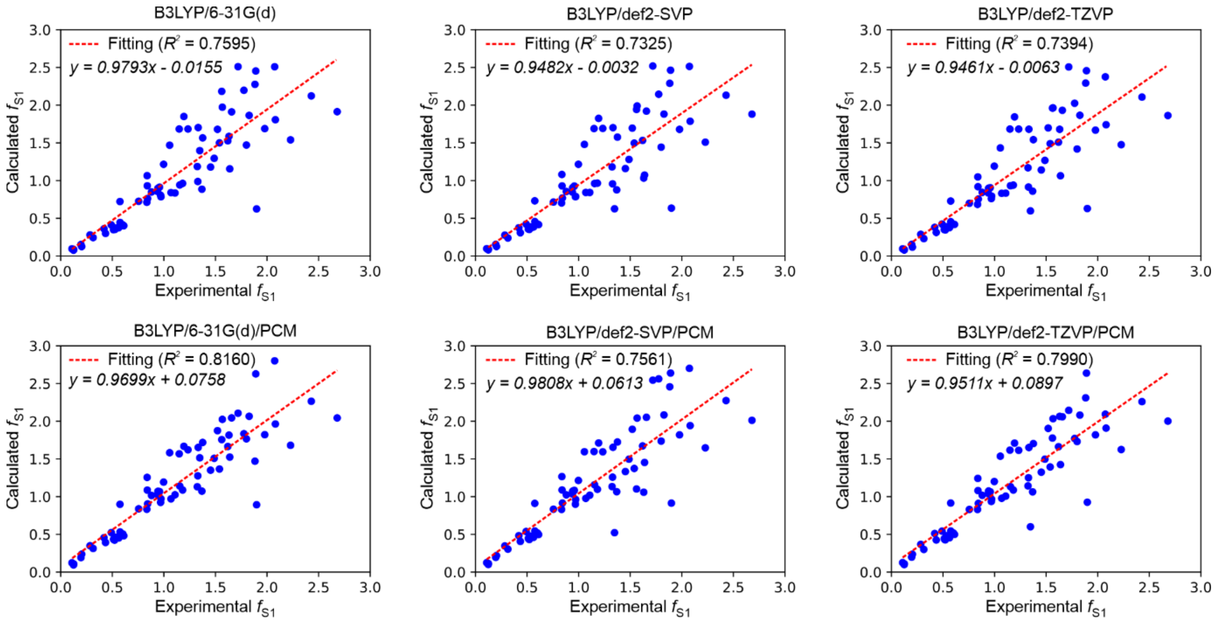

Figure S5. The correlation between the experimental fitted and calculated f_{S1} using the functional of B3LYP with different basic sets in gas phase or in solvents.

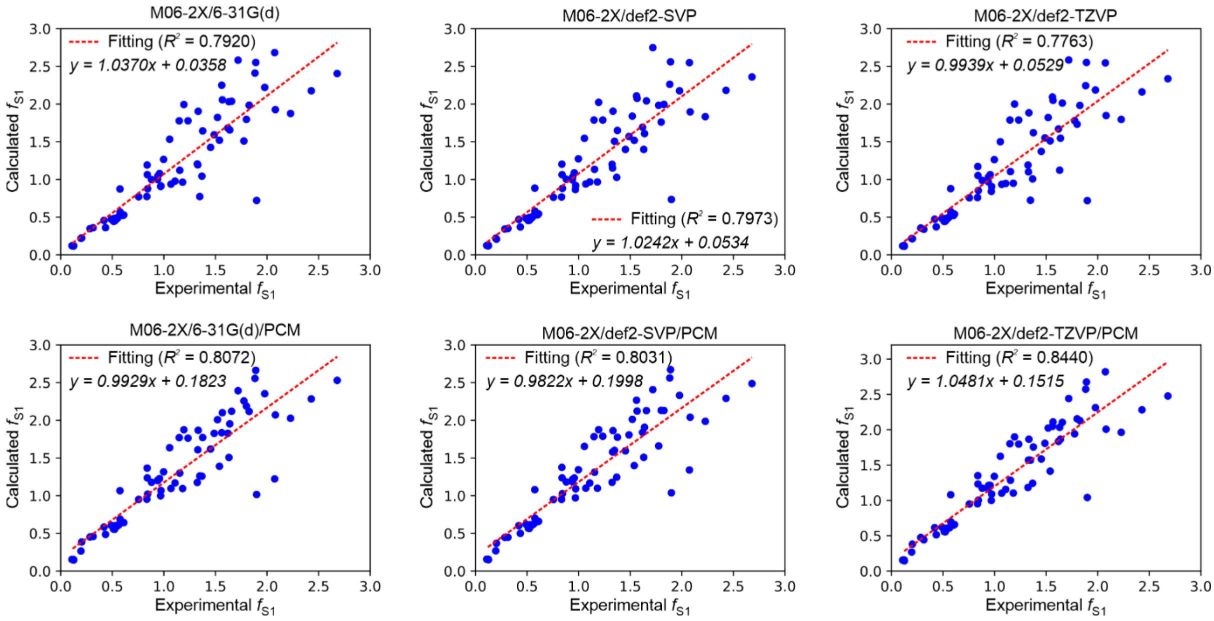

Figure S6. The correlation between the experimental fitted and calculated f_{S1} using the functional of M06-2X with different basic sets in gas phase or in solvents.

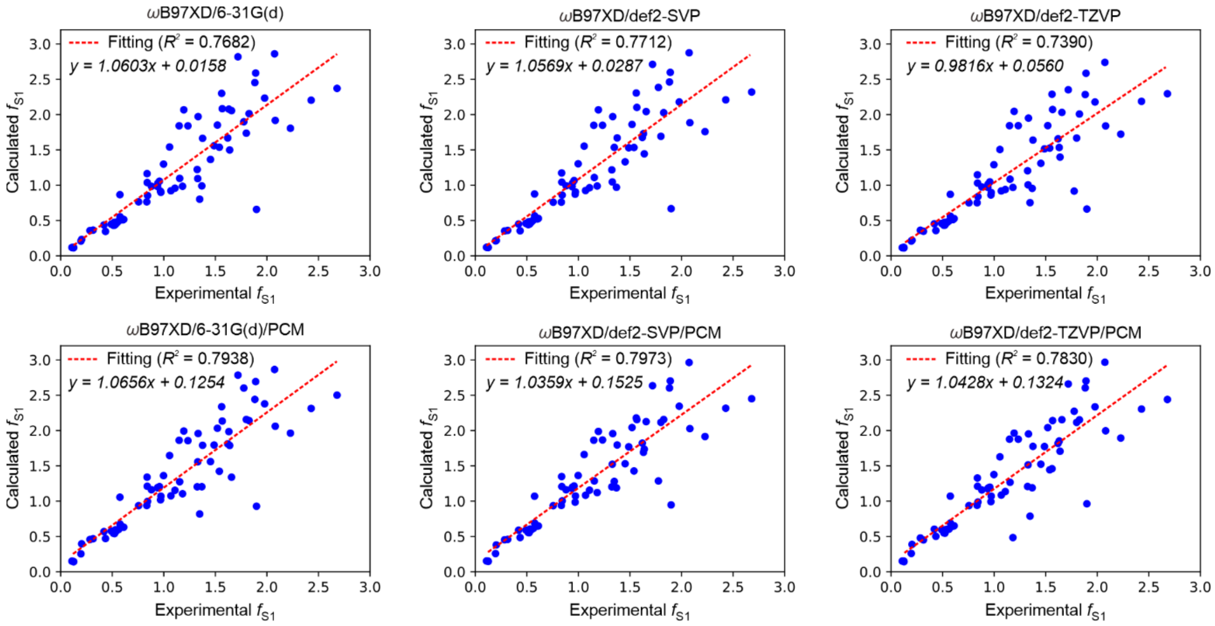

Figure S7. The correlation between the experimental fitted and calculated f_{S1} using the functional of ωB97XD with different basic sets in gas phase or in solvents.

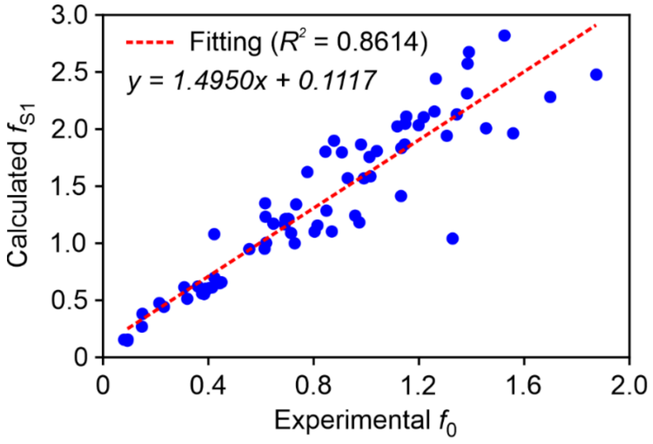

Figure S8. The correlation between the experimental fitted f_0 and calculated f_{S1} using M06-2X/def2-TZVP/PCM.

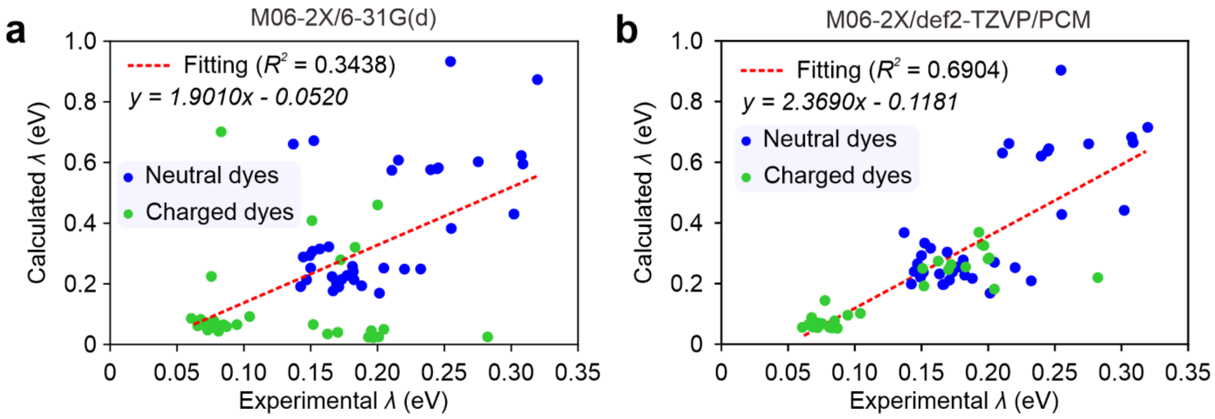

Figure S9. The correlation between the experimental fitted and calculated reorganization energy using (a) M06-2X/6-31G(d) and (b) M06-2X/def2-TZVP/PCM, highlighting the charged dyes.

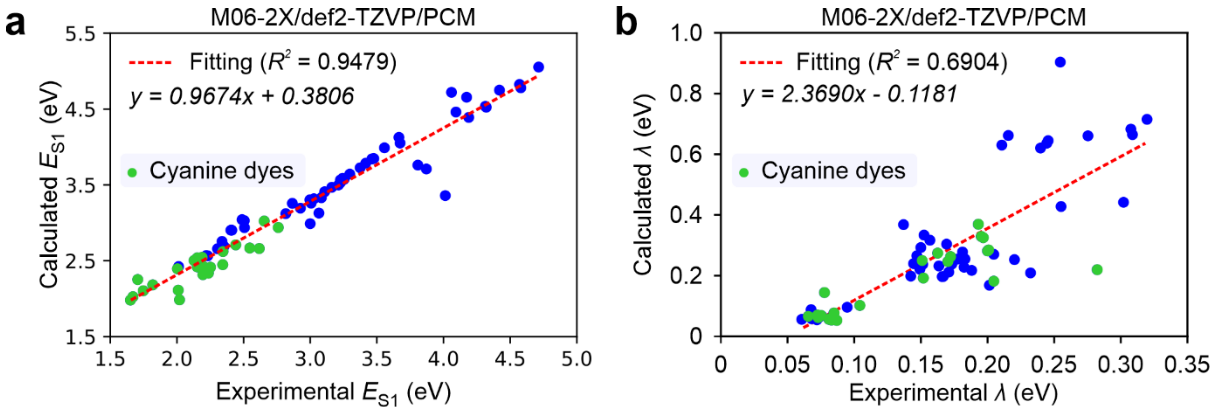

Figure S10. The correlation between the experimental fitted and calculated (a) E_{S1} and (b) reorganization energy using M06-2X/def2-TZVP/PCM, highlighting the cyanine dyes.

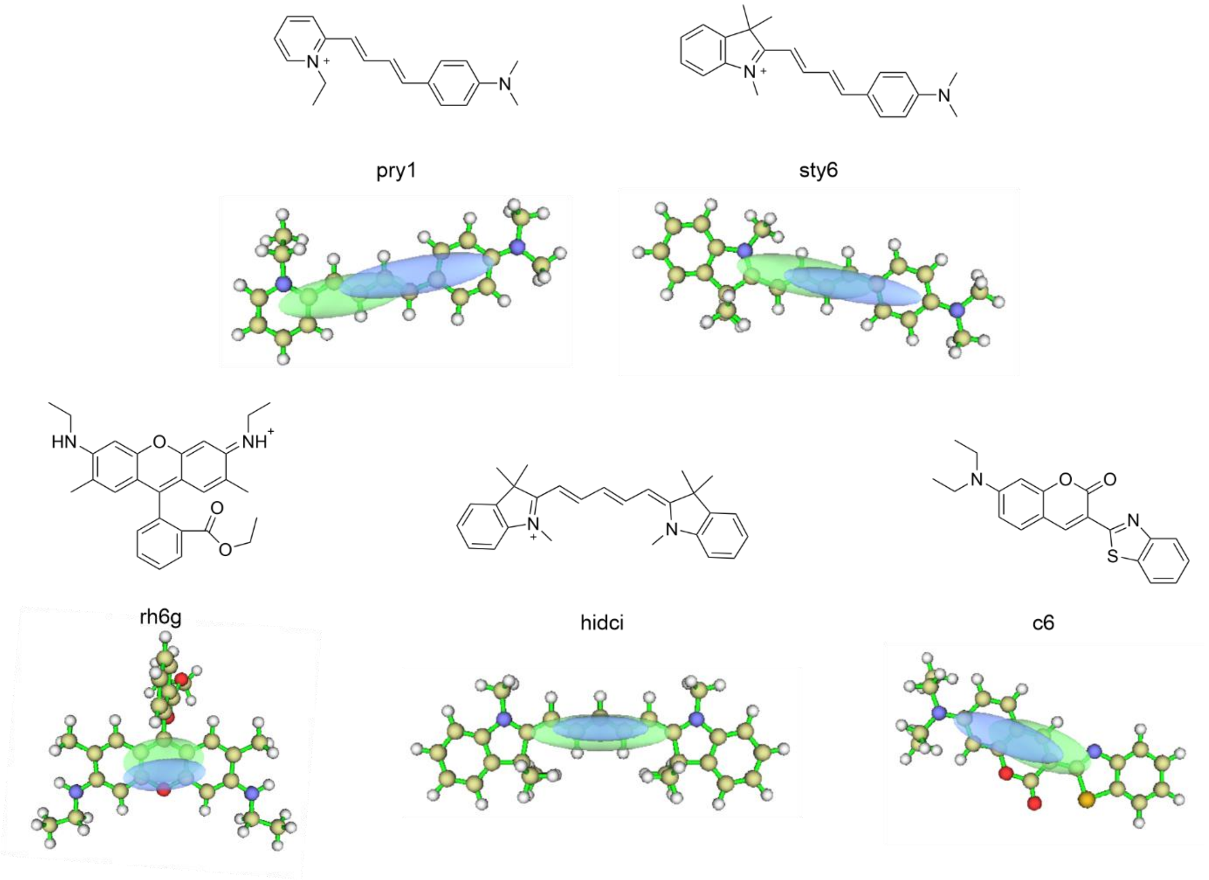

Figure S11. The distribution of electron and hole of **rh6g**, **hidci**, **sty6**, **pry1**, and **c6** using M06-2X/def2-TZVP/PCM. The green region represents the electron distribution, and the blue region represents the hole distribution.

Table S3. The distance between the centroid of the electron and hole of **rh6g**, **hidci**, **sty6**, **pry1**, and **c6** in the Frank Condon state.

Dyes	Charge	Experimental λ (eV)	M06-2X/6-31G(d)		M06-2X/def2-TZVP/PCM	
			D_{CT} (Å)	Calculated λ (eV)	D_{CT} (Å)	Calculated λ (eV)
rh6g	+1	0.068	0.844	0.083	0.818	0.088
hidci	+1	0.072	0.150	0.065	0.213	0.070
sty6	+1	0.205	2.826	0.05	2.441	0.182
pry1	+1	0.195	3.514	0.046	3.702	0.329
c6	0	0.166	0.927	0.224	1.719	0.197

Table S4. Calculated ZPE in the S_0 and S_1 state and their ΔZPE for sample molecules using M06-2X/def2-TZVP/PCM.

Dyes	ZPE_{S0} (eV)	ZPE_{S1} (eV)	ΔZPE_{S1-S0} (eV)
061	3.461	3.385	-0.076
068	4.029	3.975	-0.054
c6	9.346	9.277	-0.069
c102	8.266	8.177	-0.089
daspi	9.503	9.447	-0.056
dqoci	11.242	11.192	-0.050
hidci	14.106	14.076	-0.030
pheox	9.292	9.245	-0.046
pry1	10.420	10.382	-0.038
rh6g	14.693	14.645	-0.048
rh110	8.526	8.483	-0.043
sty6	12.270	12.231	-0.039

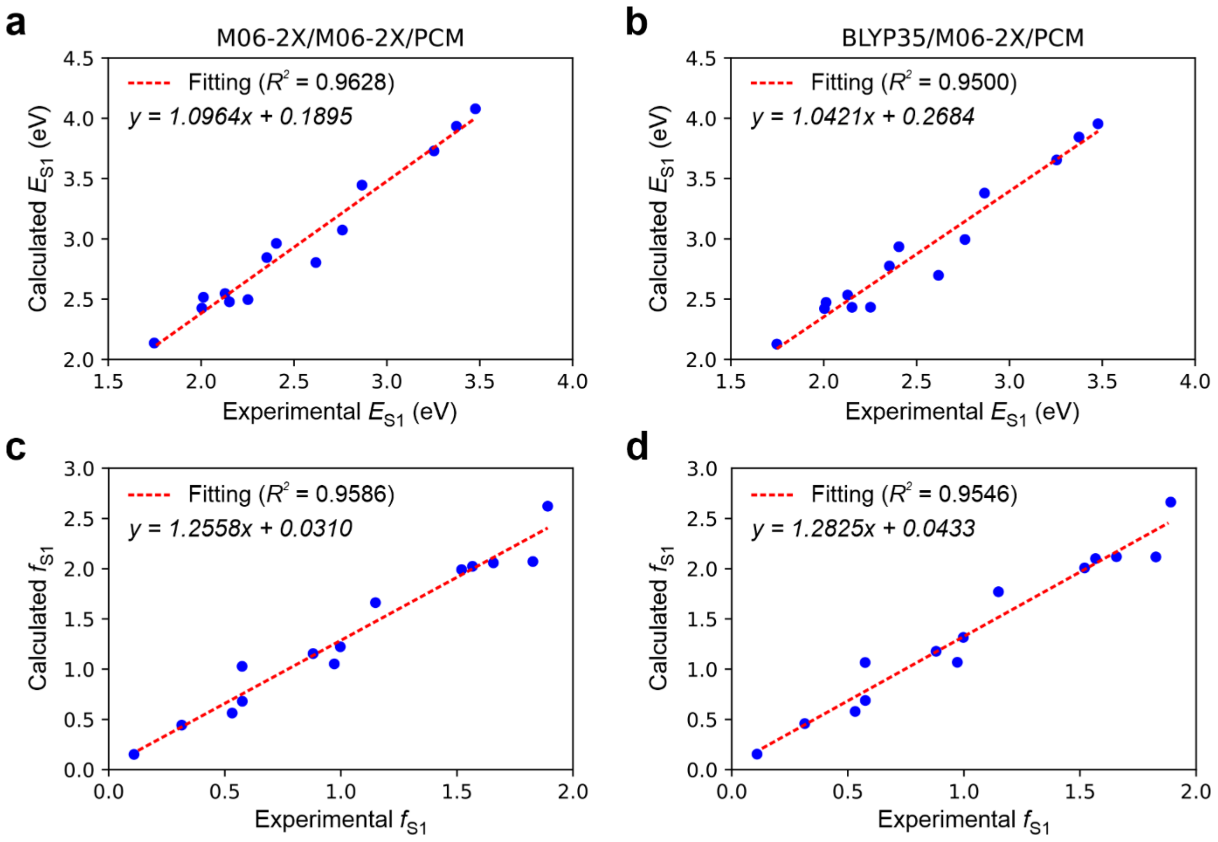

Figure S12. The correlation between the experimental fitted and calculated (a) E_{S1} and (c) f_{S1} using BLYP35/6-31G(d)/PCM for the optimization of ground state and M06-2X/6-31G(d)/PCM for the TDDFT calculations. The correlation between the experimental fitted and calculated (a) E_{S1} and (c) f_{S1} using M06-2X/6-31G(d)/PCM for the optimization of ground state and the TDDFT calculations. We selected 14 molecules as samples. The results from the two different systems show minimal differences; however, the computational cost of the optimization process using BLYP35/6-31G(d)/PCM is approximately half that of M06-2X/6-31G(d)/PCM.

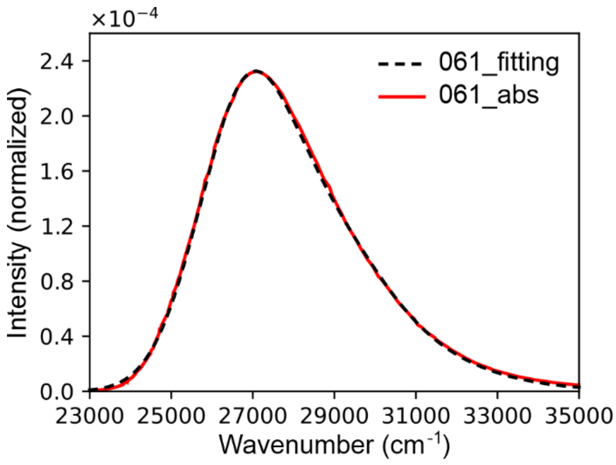

Figure S13. The experimental absorption and fitting spectra of 061.

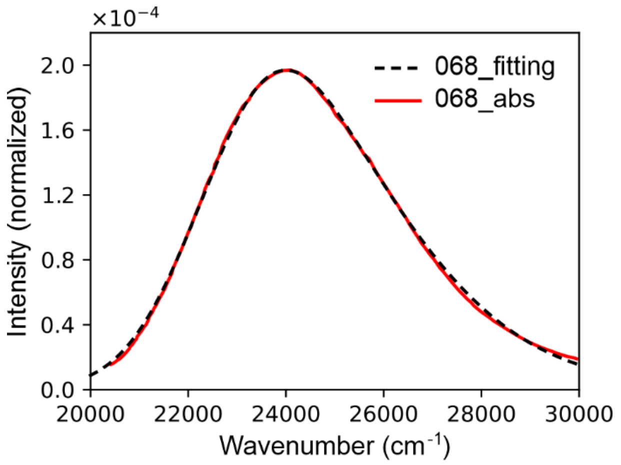

Figure S14. The experimental absorption and fitting spectra of 068.

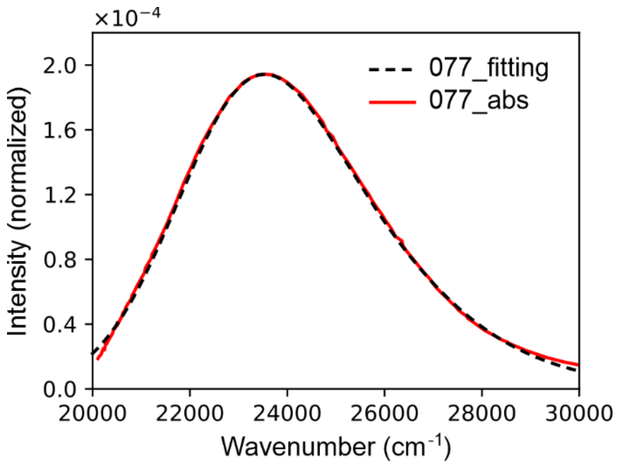

Figure S15. The experimental absorption and fitting spectra of 077.

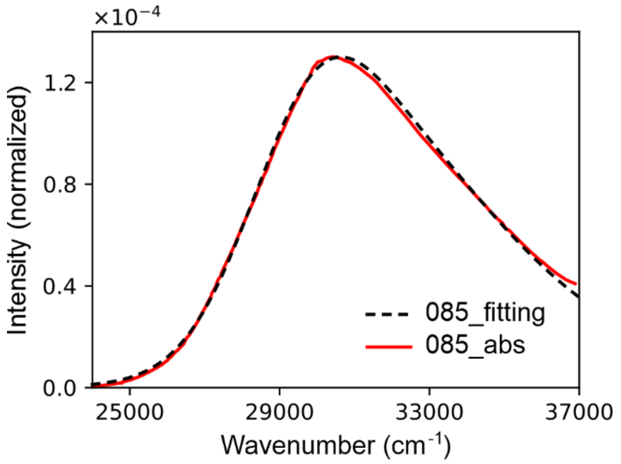

Figure S16. The experimental absorption and fitting spectra of 085.

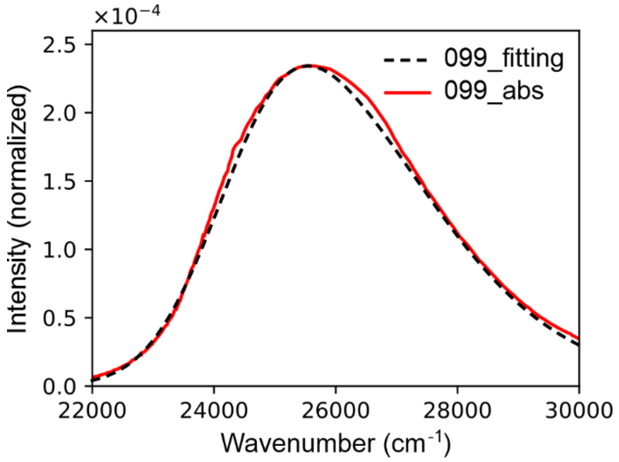

Figure S17. The experimental absorption and fitting spectra of 099.

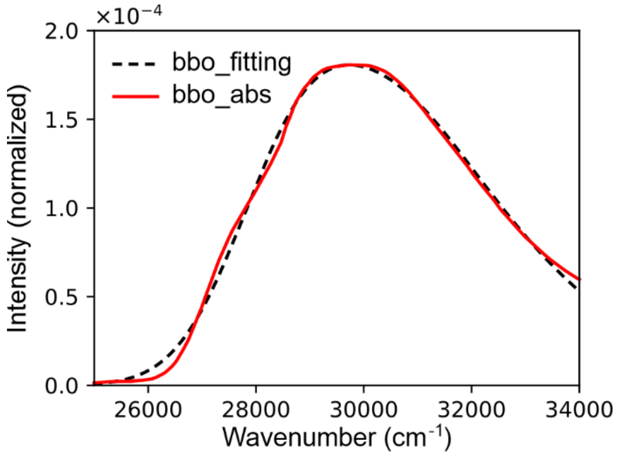

Figure S18. The experimental absorption and fitting spectra of bbo.

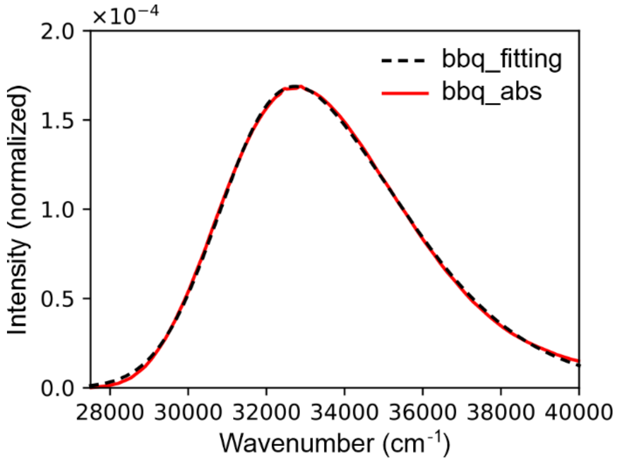

Figure S19. The experimental absorption and fitting spectra of bbq.

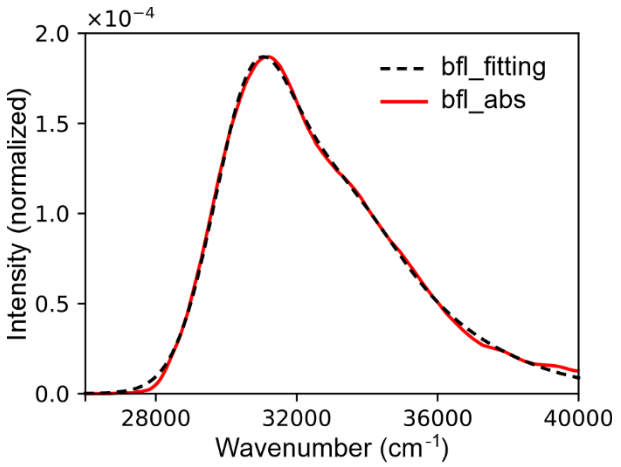

Figure S20. The experimental absorption and fitting spectra of bfl.

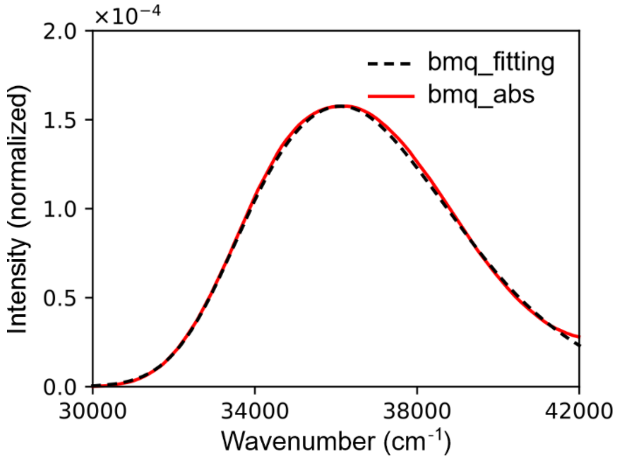

Figure S21. The experimental absorption and fitting spectra of bmq.

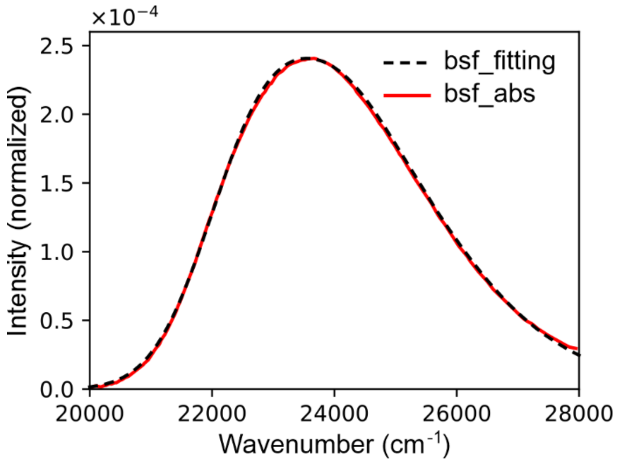

Figure S22. The experimental absorption and fitting spectra of bsf.

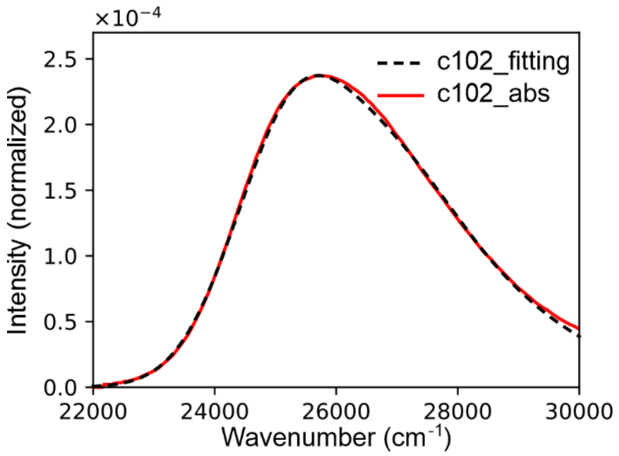

Figure S23. The experimental absorption and fitting spectra of c102.

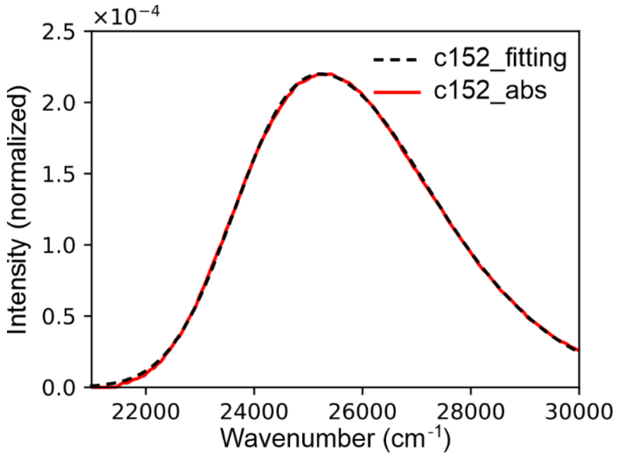

Figure S24. The experimental absorption and fitting spectra of c152.

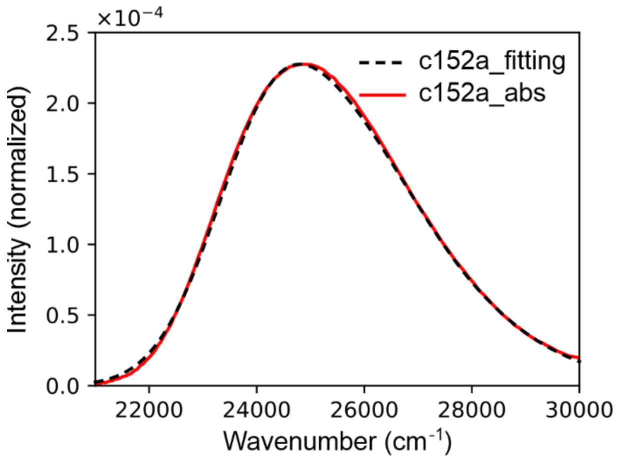

Figure S25. The experimental absorption and fitting spectra of c152a.

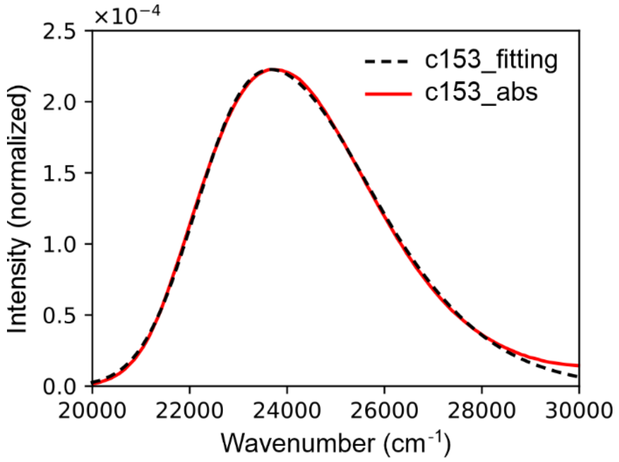

Figure S26. The experimental absorption and fitting spectra of c153.

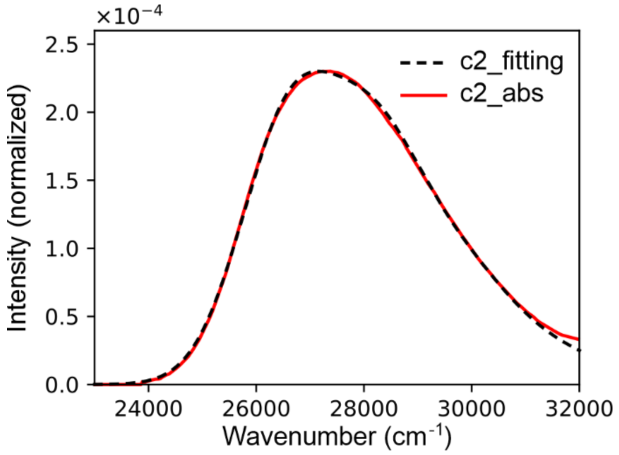

Figure S27. The experimental absorption and fitting spectra of c2.

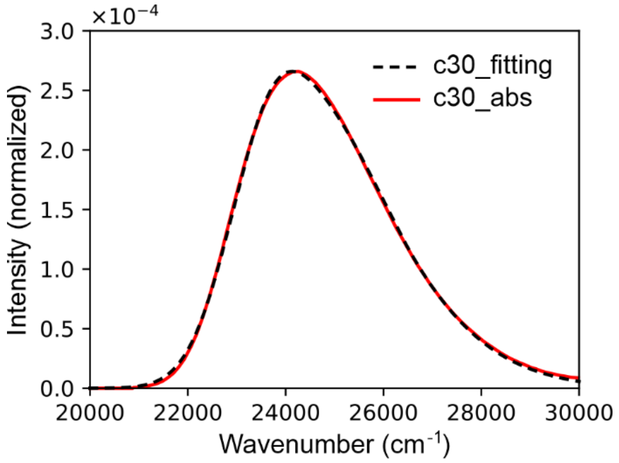

Figure S28. The experimental absorption and fitting spectra of c30.

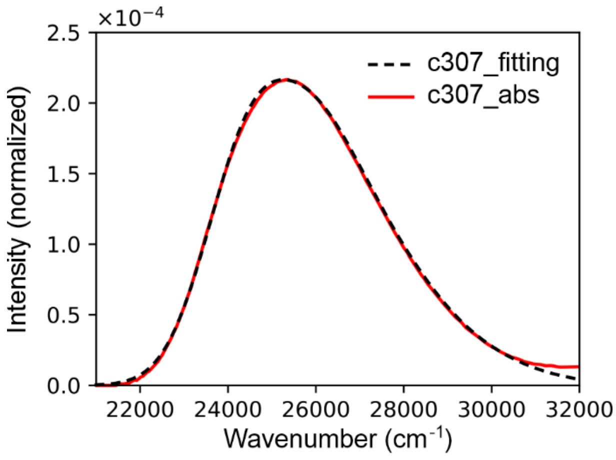

Figure S29. The experimental absorption and fitting spectra of c307.

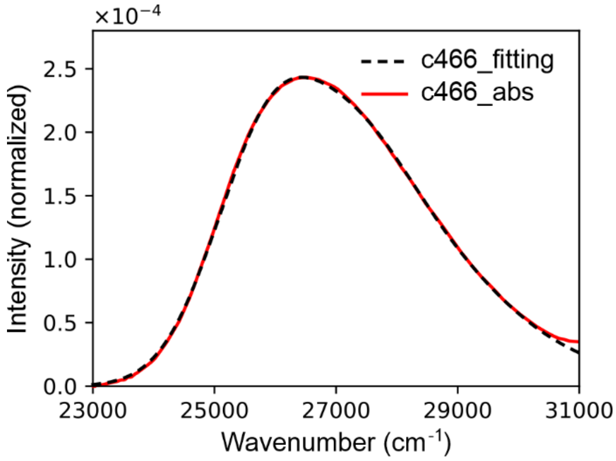

Figure S30. The experimental absorption and fitting spectra of c466.

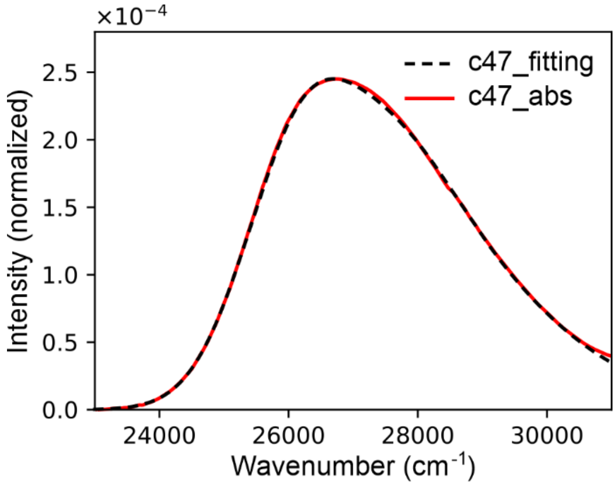

Figure S31. The experimental absorption and fitting spectra of c47.

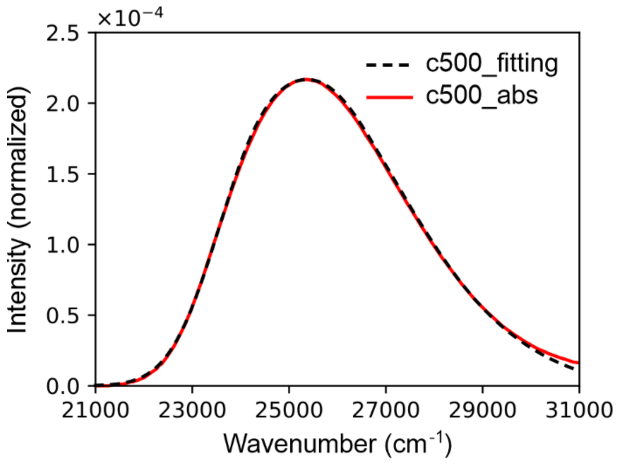

Figure S32. The experimental absorption and fitting spectra of c500.

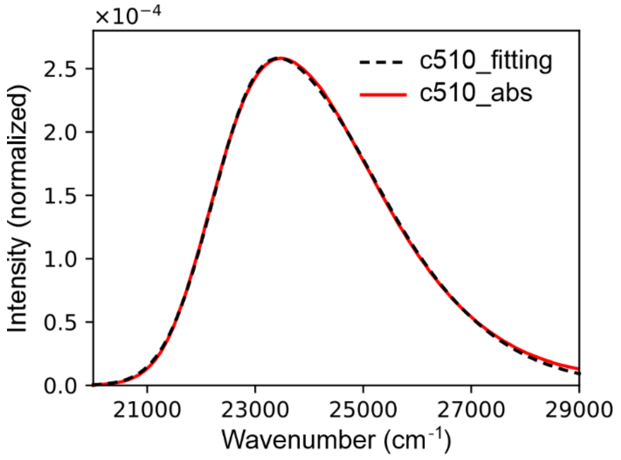

Figure S33. The experimental absorption and fitting spectra of c510.

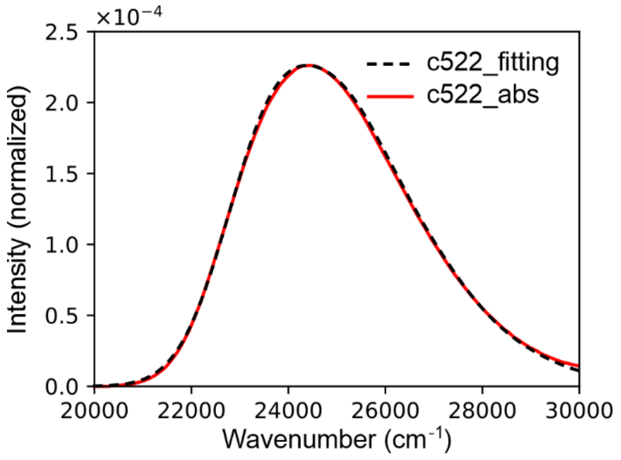

Figure S34. The experimental absorption and fitting spectra of c522.

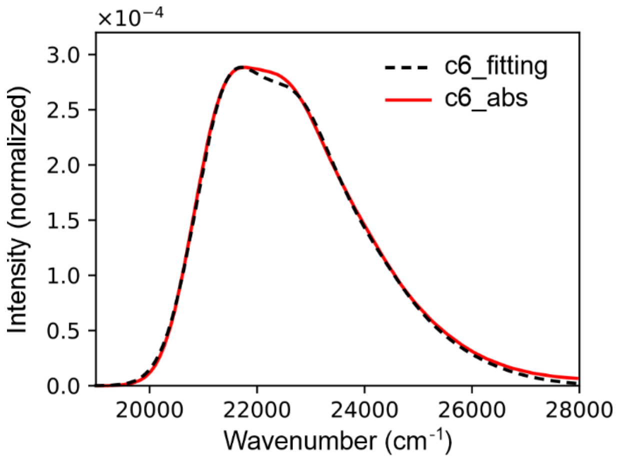

Figure S35. The experimental absorption and fitting spectra of c6.

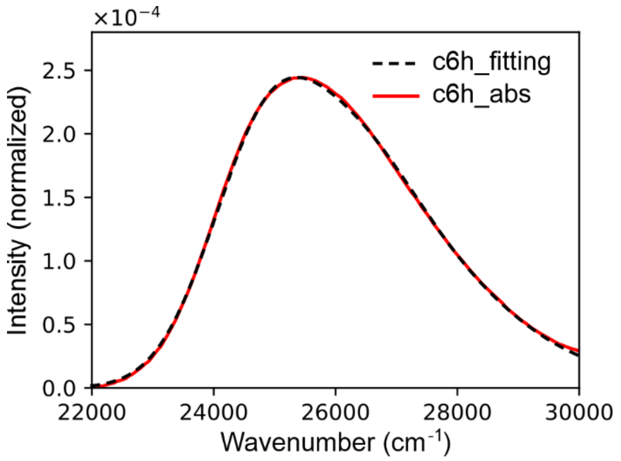

Figure S36. The experimental absorption and fitting spectra of c6h.

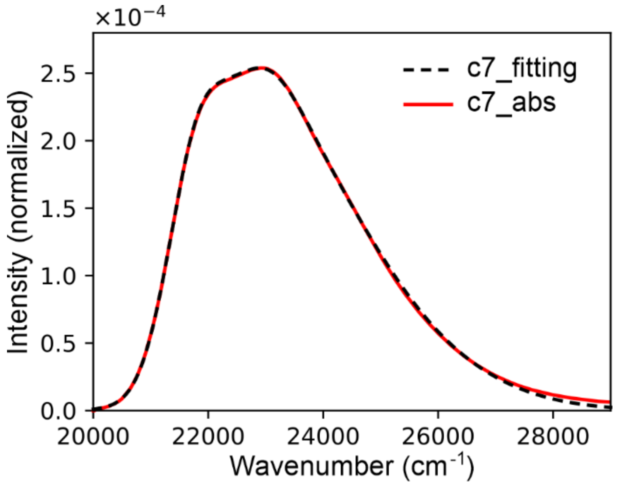

Figure S37. The experimental absorption and fitting spectra of c7.

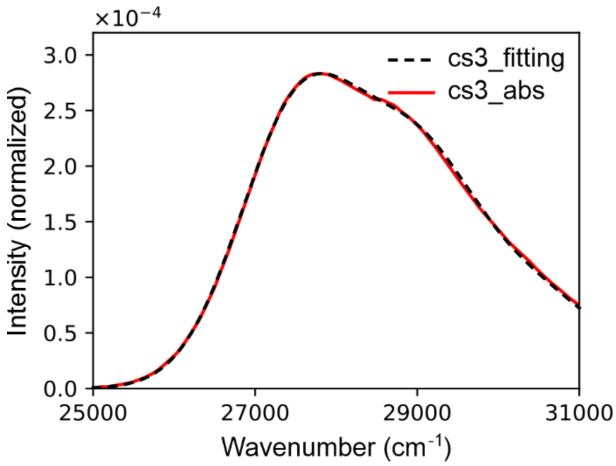

Figure S38. The experimental absorption and fitting spectra of cs3.

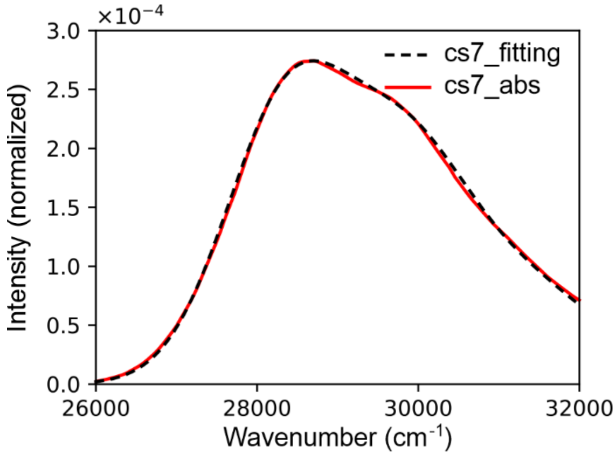

Figure S39. The experimental absorption and fitting spectra of cs7.

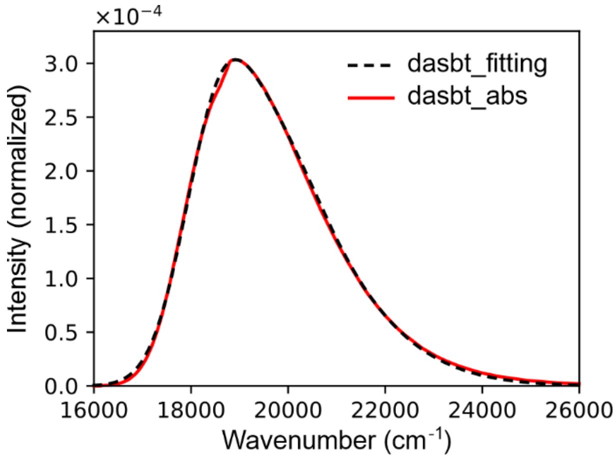

Figure S40. The experimental absorption and fitting spectra of dasbt.

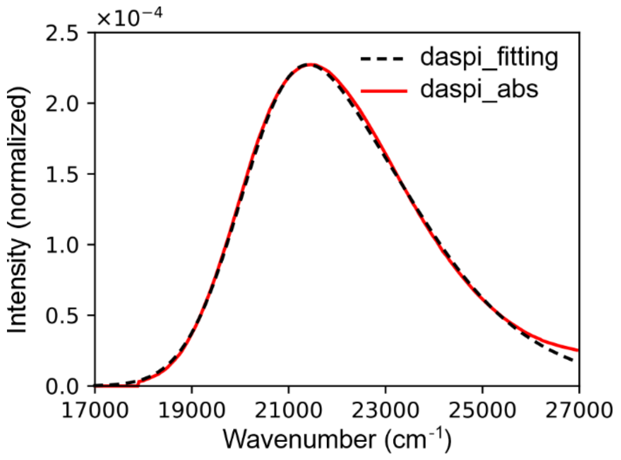

Figure S41. The experimental absorption and fitting spectra of daspi.

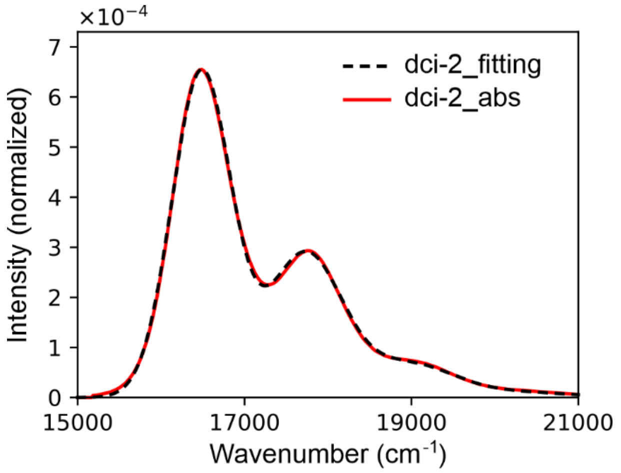

Figure S42. The experimental absorption and fitting spectra of dci-2.

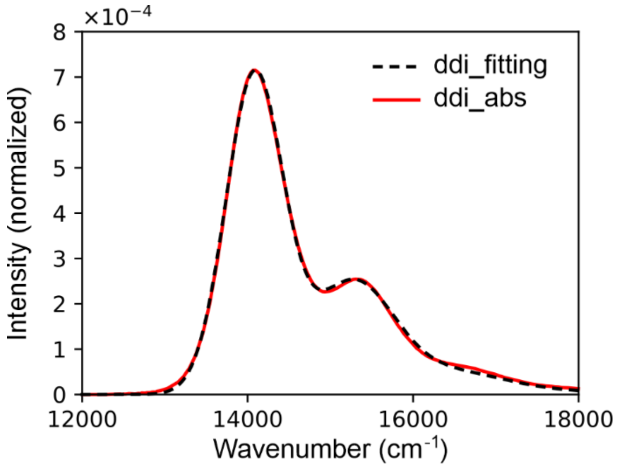

Figure S43. The experimental absorption and fitting spectra of ddi.

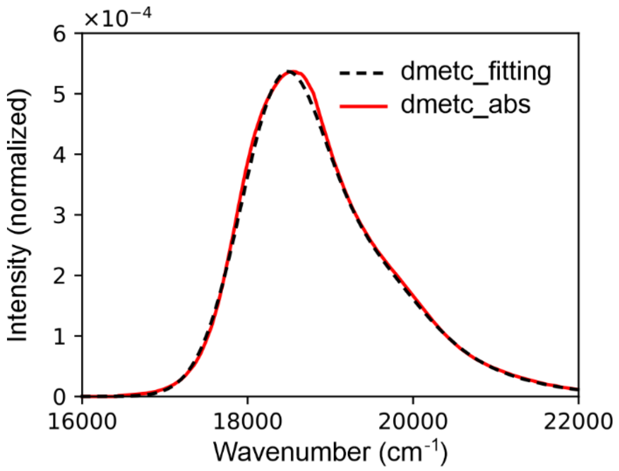

Figure S44. The experimental absorption and fitting spectra of dmetc.

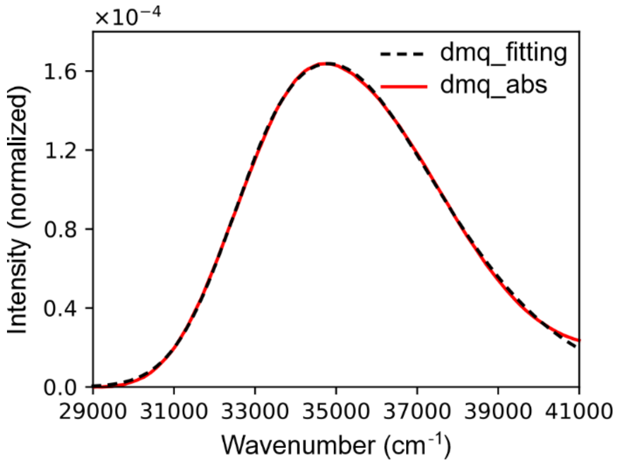

Figure S45. The experimental absorption and fitting spectra of dmq.

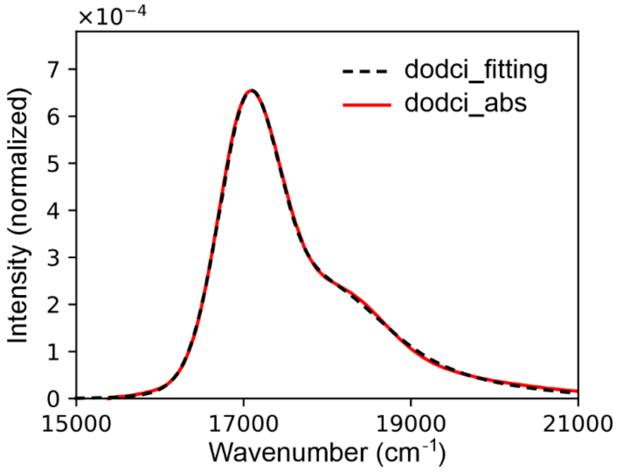

Figure S46. The experimental absorption and fitting spectra of dodci.

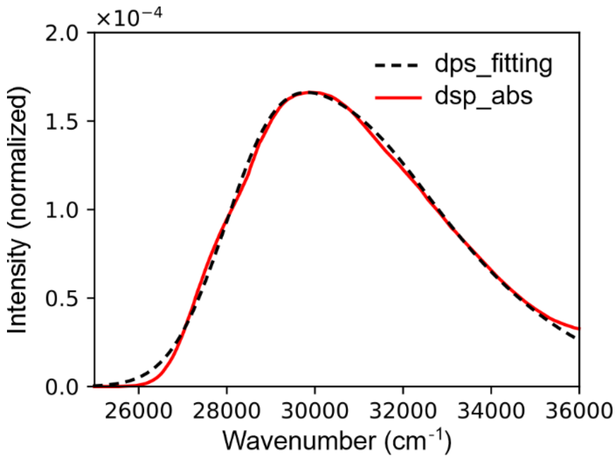

Figure S47. The experimental absorption and fitting spectra of dps.

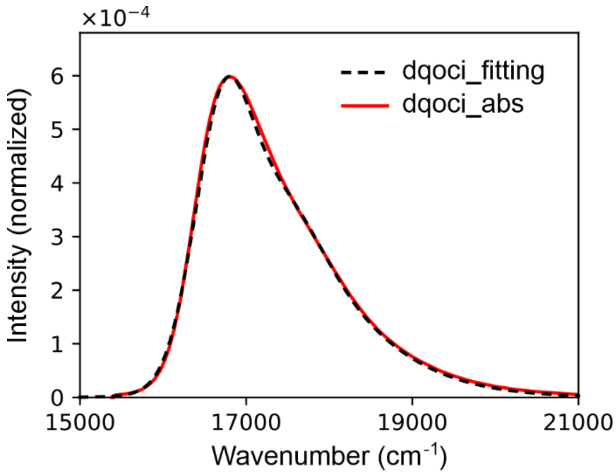

Figure S48. The experimental absorption and fitting spectra of dqoci.

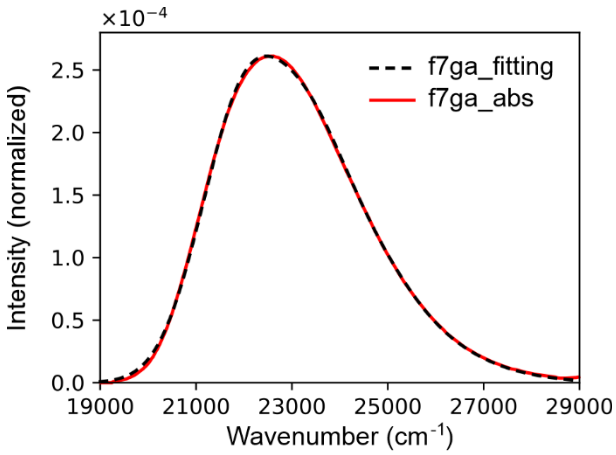

Figure S49. The experimental absorption and fitting spectra of f7ga.

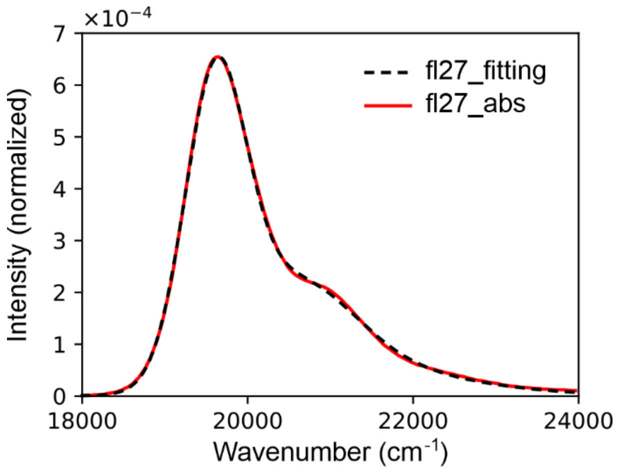

Figure S50. The experimental absorption and fitting spectra of fl27.

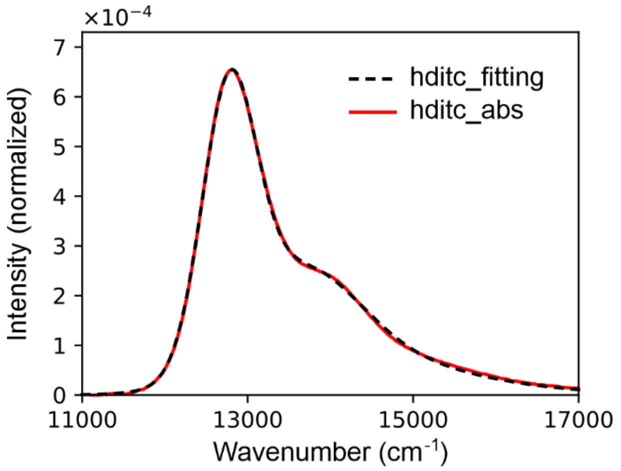

Figure S51. The experimental absorption and fitting spectra of hditc.

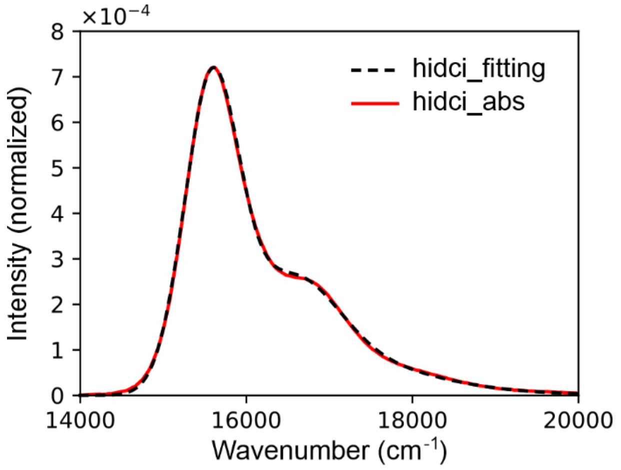

Figure S52. The experimental absorption and fitting spectra of hidci.

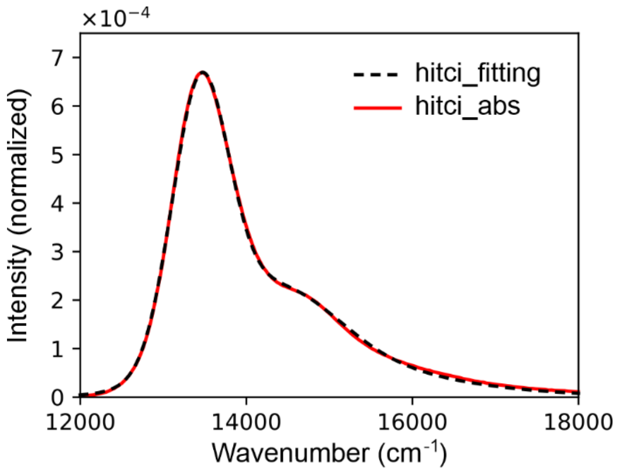

Figure S53. The experimental absorption and fitting spectra of hitci.

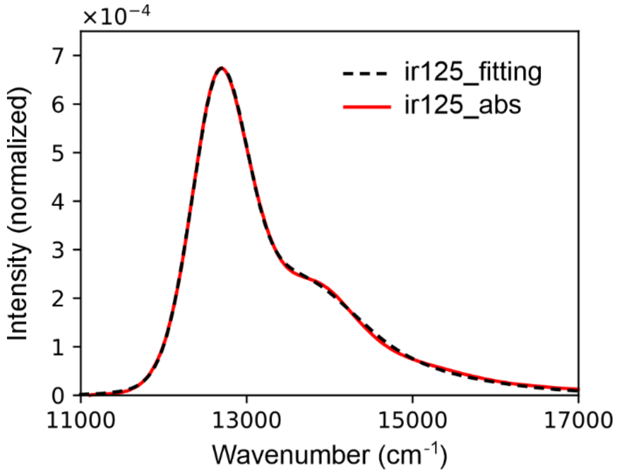

Figure S54. The experimental absorption and fitting spectra of ir125.

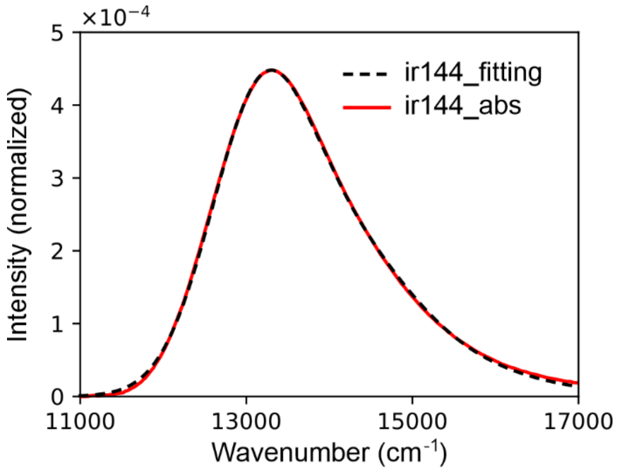

Figure S55. The experimental absorption and fitting spectra of ir144.

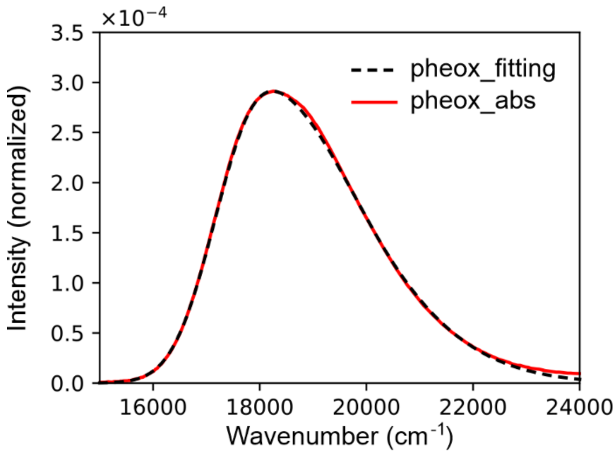

Figure S56. The experimental absorption and fitting spectra of pheox.

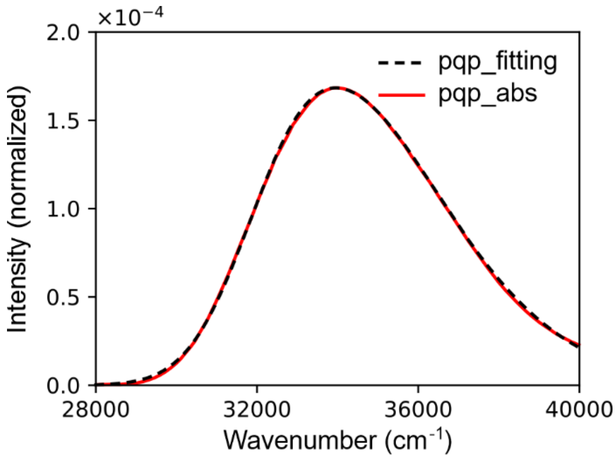

Figure S57. The experimental absorption and fitting spectra of pqp.

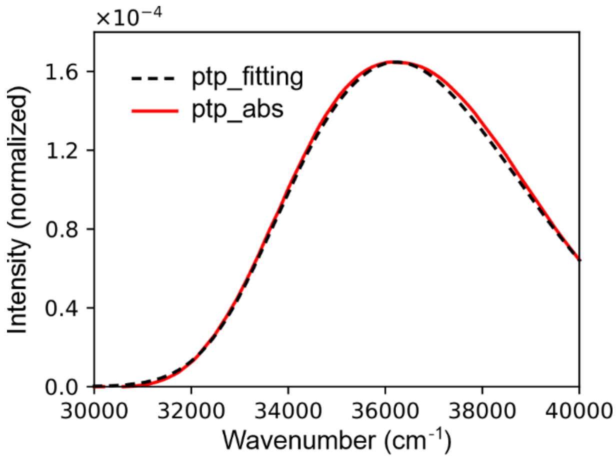

Figure S58. The experimental absorption and fitting spectra of ptp.

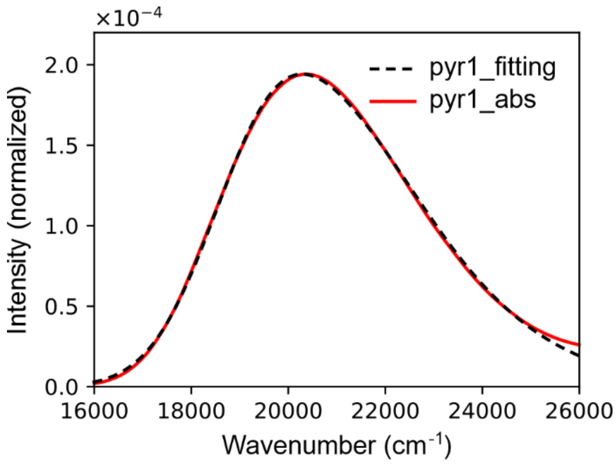

Figure S59. The experimental absorption and fitting spectra of pyr1.

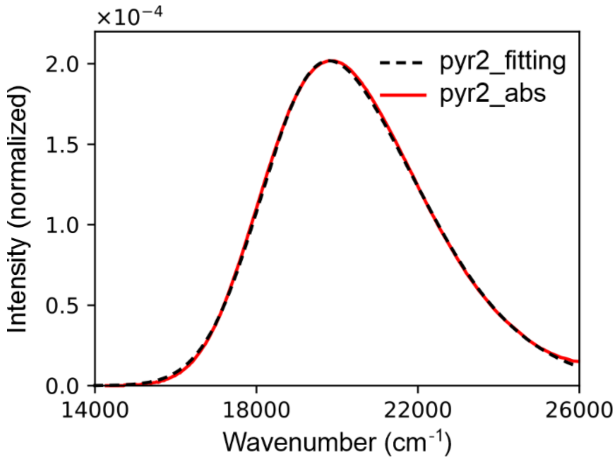

Figure S60. The experimental absorption and fitting spectra of pyr2.

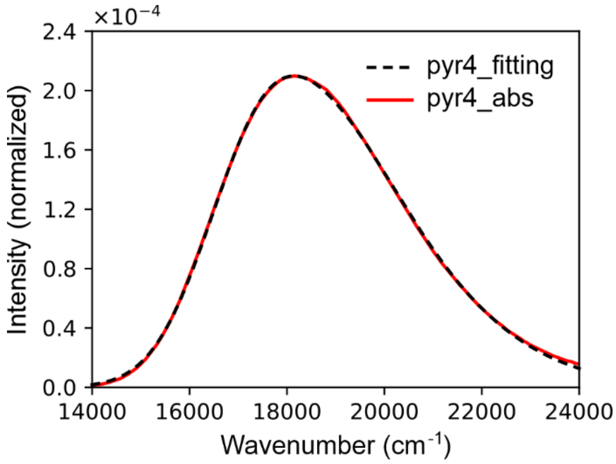

Figure S61. The experimental absorption and fitting spectra of pyr4.

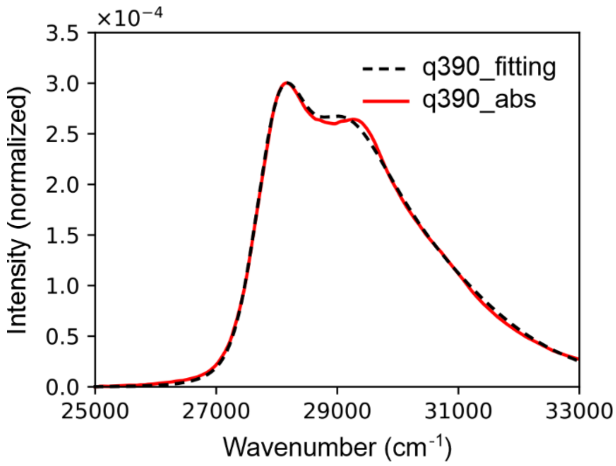

Figure S62. The experimental absorption and fitting spectra of q390.

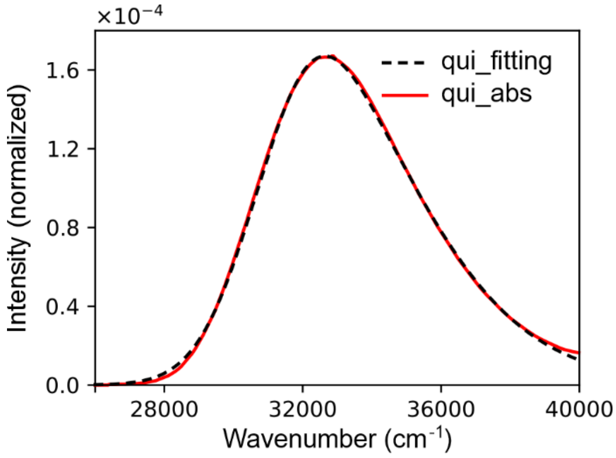

Figure S63. The experimental absorption and fitting spectra of qui.

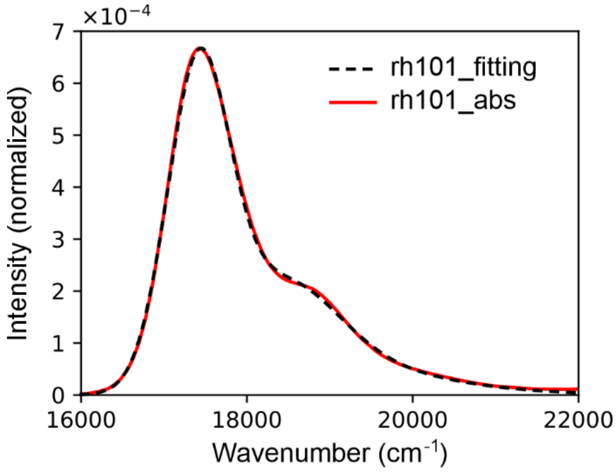

Figure S64. The experimental absorption and fitting spectra of rh101.

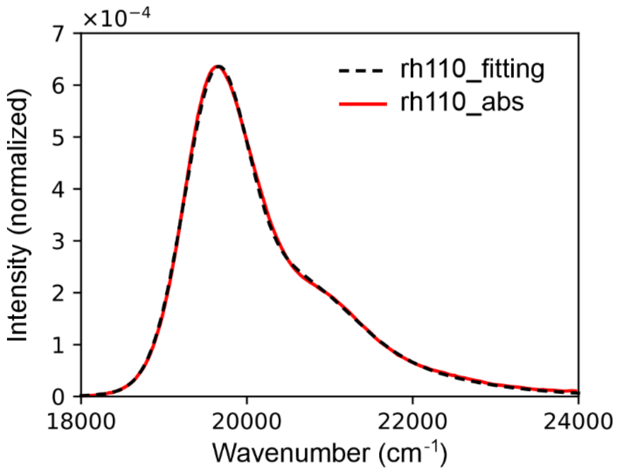

Figure S65. The experimental absorption and fitting spectra of rh110.

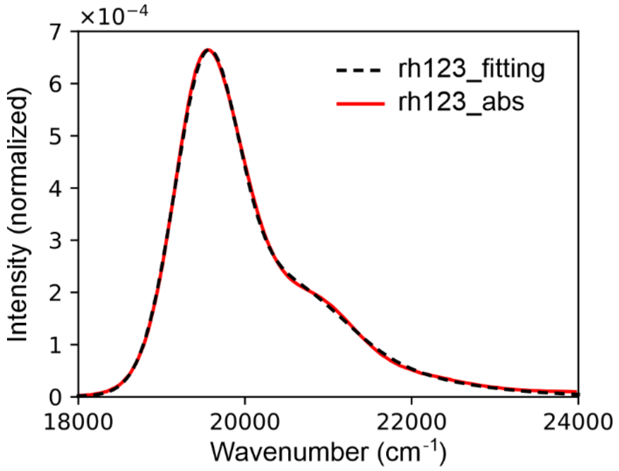

Figure S66. The experimental absorption and fitting spectra of rh123.

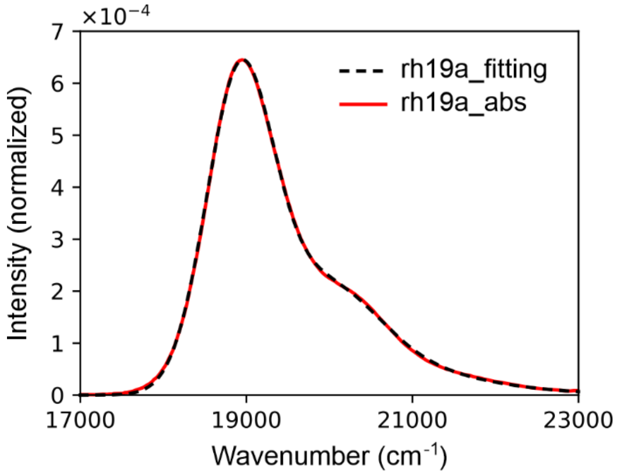

Figure S67. The experimental absorption and fitting spectra of rh19a.

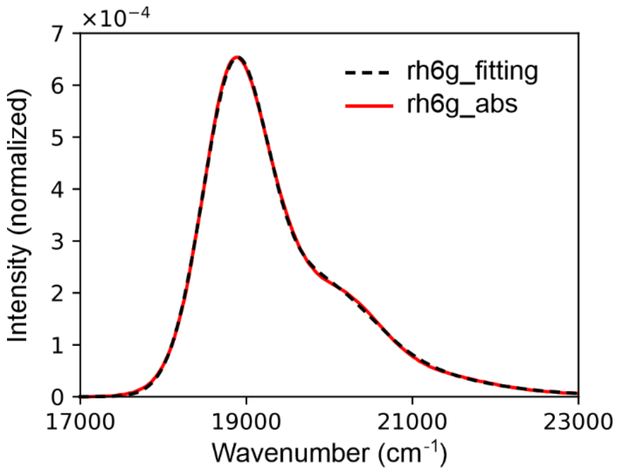

Figure S68. The experimental absorption and fitting spectra of rh6g.

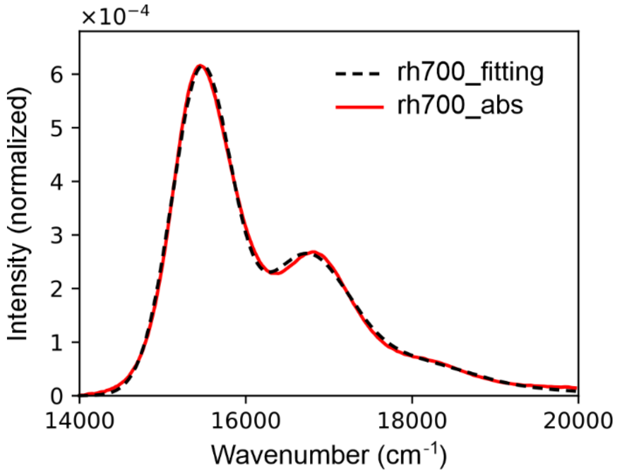

Figure S69. The experimental absorption and fitting spectra of rh700.

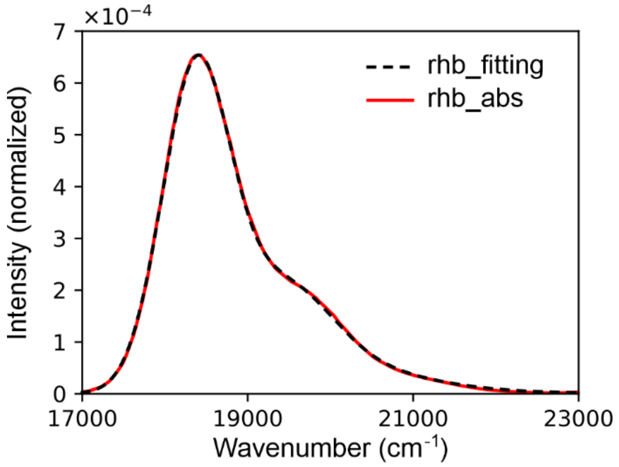

Figure S70. The experimental absorption and fitting spectra of rhb.

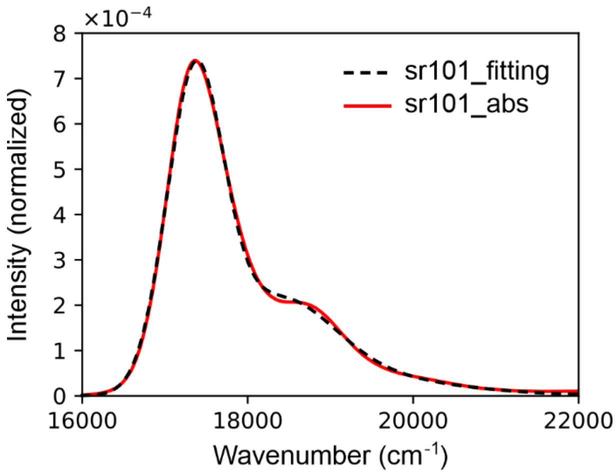

Figure S71. The experimental absorption and fitting spectra of sr101.

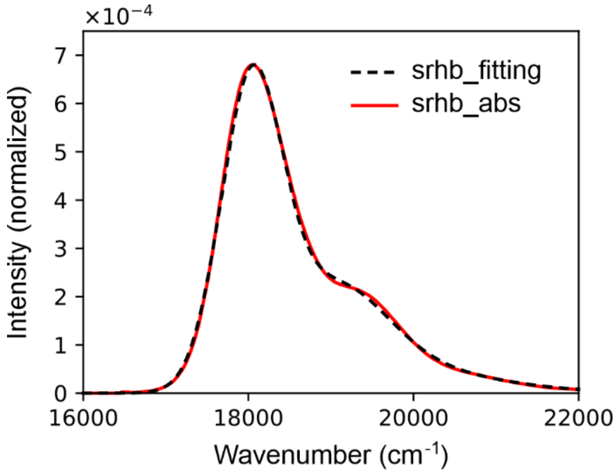

Figure S72. The experimental absorption and fitting spectra of srhb.

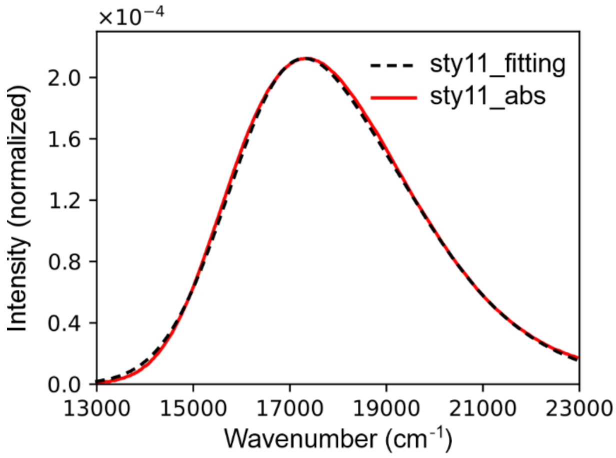

Figure S73. The experimental absorption and fitting spectra of sty11.

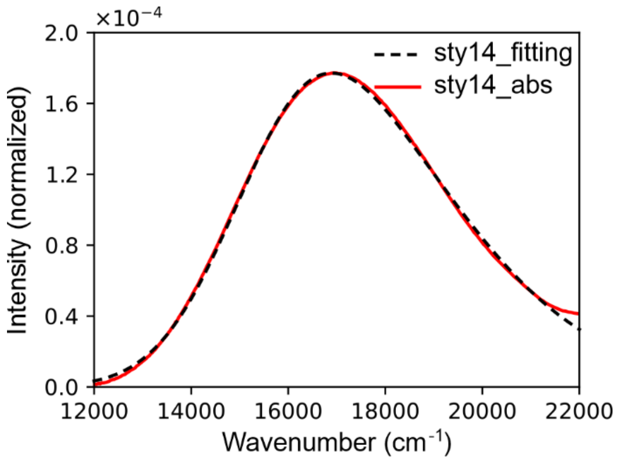

Figure S74. The experimental absorption and fitting spectra of sty14.

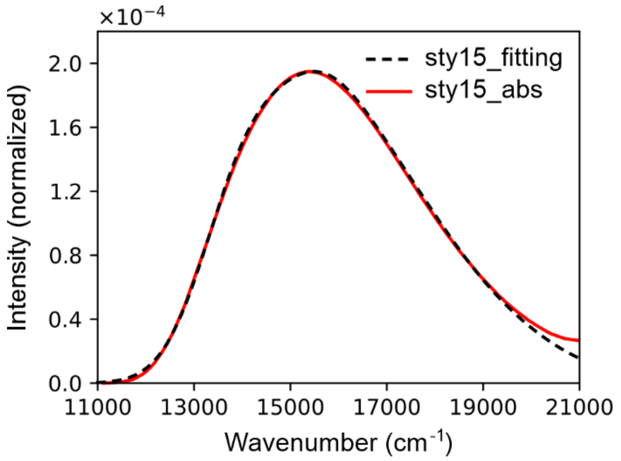

Figure S75. The experimental absorption and fitting spectra of sty15.

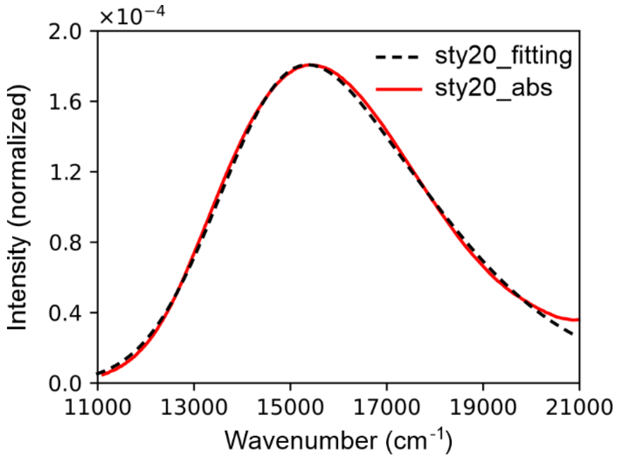

Figure S76. The experimental absorption and fitting spectra of sty20.

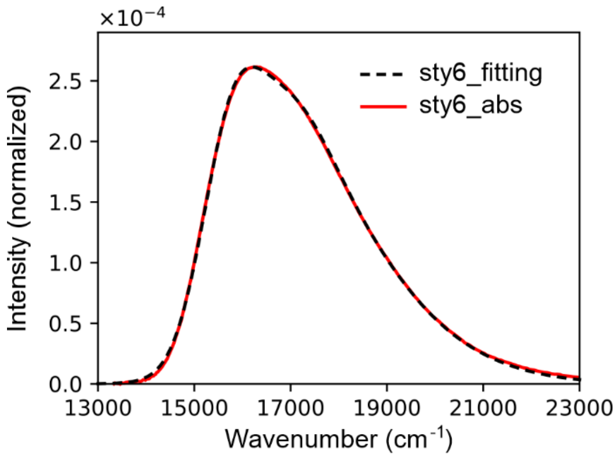

Figure S77. The experimental absorption and fitting spectra of sty6.

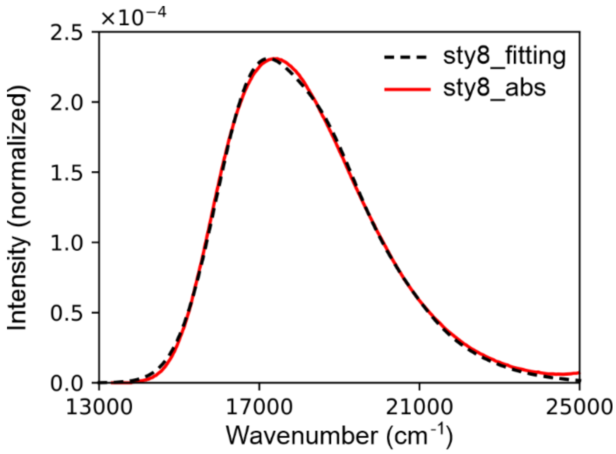

Figure S78. The experimental absorption and fitting spectra of sty8.

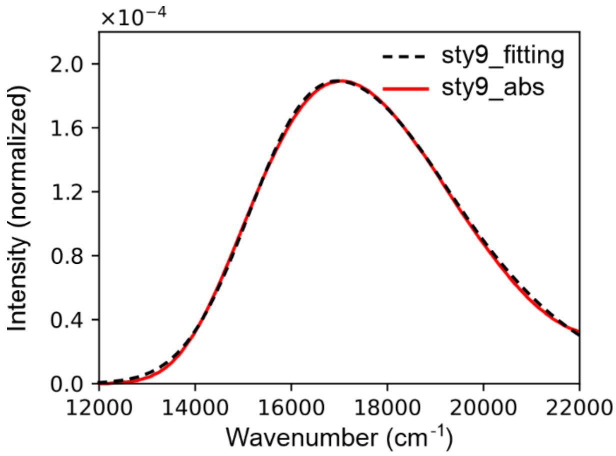

Figure S79. The experimental absorption and fitting spectra of sty9.

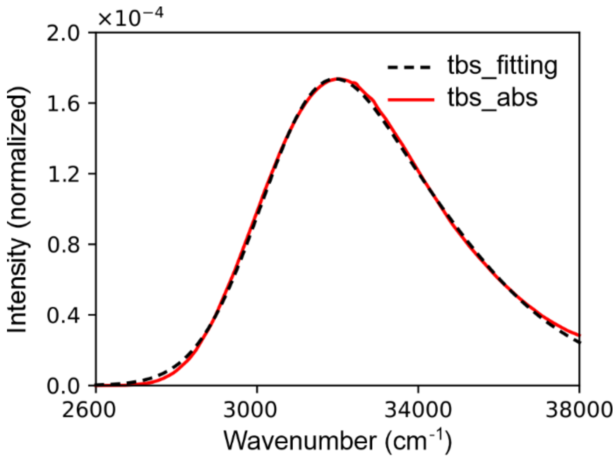

Figure S80. The experimental absorption and fitting spectra of tbs.

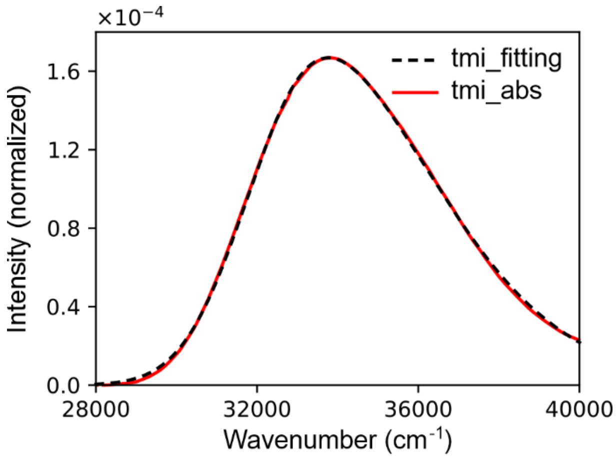

Figure S81. The experimental absorption and fitting spectra of tmi.

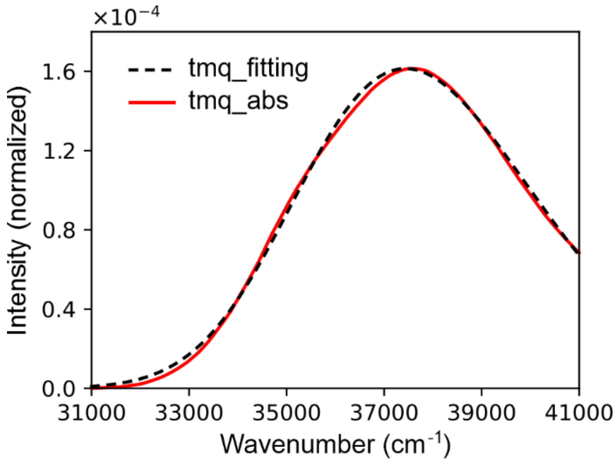

Figure S82. The experimental absorption and fitting spectra of tmq.
